# Two-dimensional nanostructures based ‘-onics’ and ‘-omics’ in personalized medicine

**DOI:** 10.1515/nanoph-2022-0439

**Published:** 2022-09-19

**Authors:** Bibi Mary Francis, Aravindkumar Sundaram, Rajesh Kumar Manavalan, Weng Kung Peng, Han Zhang, Joice Sophia Ponraj, Sathish Chander Dhanabalan

**Affiliations:** Center for Advanced Materials, Aaivalayam-DIRAC Institute, Coimbatore, Tamil Nadu, India; Institute of Natural Science and Mathematics, Ural Federal University, 620002 Yekaterinburg, Russia; Songshan Lake Materials Laboratory, Innovation Park, 523808 Dongguan, China; Key Laboratory of Optoelectronic Devices and Systems of Ministry of Education and Guangdong Province, College of Physics and Optoelectronic Engineering, Shenzhen Key Laboratory of Micro-Nano Photonic Information Technology, Guangdong Laboratory of Artificial Intelligence and Digital Economy (SZ), Institute of Microscale Optoelectronics, Collaborative Innovation Centre for Optoelectronic Science & Technology, Shenzhen University, Shenzhen 518060, China

**Keywords:** 2D materials, photonics, point-of-care technology, ‘omics’, ‘onics’

## Abstract

With the maturing techniques for advanced synthesis and engineering of two-dimensional (2D) materials, its nanocomposites, hybrid nanostructures, alloys, and heterostructures, researchers have been able to create materials with improved as well as novel functionalities. One of the major applications that have been taking advantage of these materials with unique properties is biomedical devices, which currently prefer to be decentralized and highly personalized with good precision. The unique properties of these materials, such as high surface to volume ratio, a large number of active sites, tunable bandgap, nonlinear optical properties, and high carrier mobility is a boon to ‘onics’ (photonics/electronics) and ‘omics’ (genomics/exposomics) technologies for developing personalized, low-cost, feasible, decentralized, and highly accurate medical devices. This review aims to unfold the developments in point-of-care technology, the application of ‘onics’ and ‘omics’ in point-of-care medicine, and the part of two-dimensional materials. We have discussed the prospects of photonic devices based on 2D materials in personalized medicine and briefly discussed electronic devices for the same.

## 1 Introduction

One of the most significant developments in healthcare in the last few decades is the decentralization of diagnosis and prognosis and their personalization to the point of care. This revolution in personalized care is primarily due to the development of a vast number of low-cost, faster, and user-friendly devices [1–3] such as sensors and lab-on-a-chip; genomic technologies such as gene editing [4, 5], 3D genomics [6, 7], functional genomics [8], and epigenomics [9]; and knowledge on exposomics which is a measure of the impact of a human’s lifestyle and lifetime exposure to environment on their health. These devices and systems are developed by combining knowledge of ‘-omics’ (genomics and exposomics) and ‘-onics’ (electronics and photonics), that can lead to precise biomarkers of health and disease [10].

Advances in ‘onics’ have led to the miniaturization of devices such as the NMR system with smaller electronic consoles, probes, and microfluidic-based chips, improving its application in point-of-care medical diagnosis [11, 12]. The two layers of ‘omics’ – genomics, and exposomics have furthered processes such as profiling tumor cells, DNA, and RNA, phenotyping diabetes mellitus, and detecting various pathological states [11]. Additionally, these developments have revolutionized the field of clinical diagnostics with artificial intelligence-based image analysis [13] and the development of wise, connected PoC devices based on the Internet of Things (IoT) [14].

A critical factor in the efficiency of PoC healthcare is the characteristics of the materials used in the devices deployed for diagnosis and prognosis. Here, nanomaterials have an advantage. Compared to conventional silicon-based semiconductor devices, nanomaterials-based devices are compact, low-cost, more sensitive, faster, and lighter [15]. Furthermore, the typical size of nanomaterials match the size of the components of living organisms, enabling effective interaction between devices and bio systems [2]. Nanomaterials like carbon nanotubes and silicon nanowires have added the advantage of good tunability (from diameter dependence of bandgap) [16]. It has to be emphasized that the distinct advantage of nanomaterials-based devices over conventional biomedical methods is in sensitivity – which means minimal false negatives [2, 17]. However, disparities in dimensions and alignment constrain the use of carbon nanotubes and silicon nanowires in conventional device fabrication. This, along with the need for more sensitivity, accuracy, biocompatibility, and reliability, kept pushing researchers to find better alternatives [18, 19]. 2D materials - another class of nanomaterials such as graphene, transition metal dichalcogenides (TMDs), MXenes, and hexagonal boron nitride (hBN) are proving to be more effective in biomedical applications [20].

Advanced synthesis and engineering of 2D materials allow us to create various functionalities via defect engineering, chemical/molecular doping [21–24], and synthesis of heterostructures, nanocomposites, or alloys with other nanomaterials [25–27]. Thus, functionalized 2D materials have a significant role in building *in vitro* and *in vivo* diagnostic sensors and imaging devices for protein transducers, drug delivery vehicles, and diagnosis of microbes and cancer cells [2].

**Figure 1:**
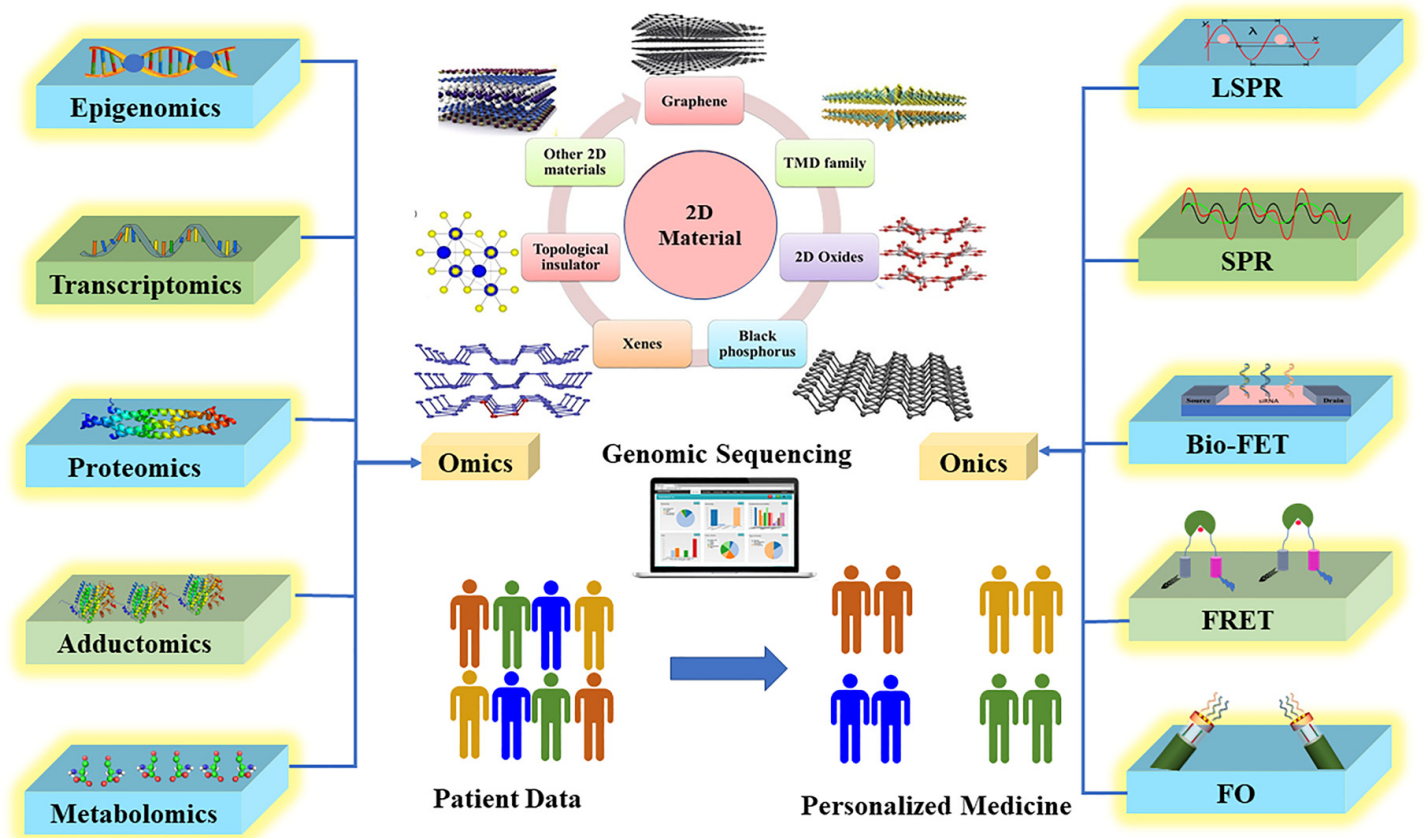
Schematic representation of review oraganization.

This review discusses the role and scope of 2D materials in ‘onics’ and ‘-omics’-based technologies in personalized medicine. As depicted in Figure 1, we start with our view on point-of-care technology (PoCT) - its significance, advantages, developments, and obstacles. We describe ‘-omics’ and ‘-onics’ technologies and the relevance of their union in PoCT. We have also explained the conventional materials used in ‘onics’ technology and their limitations. We clearly demonstrate the complimenting state of 2D materials and the importance of integrating them in PoCT. The scope of 2D materials in ‘onics’ for PoCT is discussed in detail.

## 2 Point of care technology (PoCT)

PoC personalized medicine has the potential to develop highly responsive therapies for various diseases. It considers an individual’s unique genetics and exposome and aims at targeted diagnosis, prognosis, and treatment rather than a generalized one [28]. With the support of technology, point-of-care medicine has come a long way with PoC devices that are user-friendly, low-cost, and miniaturized with reduced turnaround time (time between sample collection and analysis) [1]; they enable personalized and decentralized preventive medical screening resulting in patient-specific and timely treatment [1, 29], [30], [31], [32]. PoC technology, at present, includes devices ranging from the commonly used blood-glucose testers to viscoelastic coagulation assays [1]. These devices enable constant monitoring of physical conditions (e.g., blood sugar, blood pressure, or stress) and automated data processing, resulting in early detection of diseases [14].

The advances in genomics have enabled the PoCT since the beginning, that in the 1990s [3, 33]. Understanding human genome sequences and developing tools, sophisticated statistics, and computational methods have led to the identification of many human diseases and the realization of genomic medicine [34, 35]. Genomic medicine uses a patient’s genomic information to assess the individual’s or his family’s risk for a particular disease, diagnose rare diseases, and improve medicine efficacy [36, 37]. Genomic analysis of cancer has enabled the development of personalized therapeutic agents [38]. A new paradigm, ‘exposome,’ sums up a person’s exposure to micro- (e.g., microbiomes) and macro-environment (e.g., pollution, lifestyle) and complements the concept of the genome. Exposome has a significant impact and greater attributable risk on human health [3, 39]. Environmental factors such as air pollution and a person’s lifestyle can significantly develop various chronic pathologies, including respiratory diseases and diabetes mellitus [39]. This idea that an individual’s environment dramatically influences their traits [37] has overhauled the hypes associated with personalized disease stratification and prevention, which had depended solely on genomic medicine established on the molecular basis of health and disease [3]. This understanding has been advantageous in developing agents that could target patient groups for whom traditional health care has failed [29].

**Figure 2:**
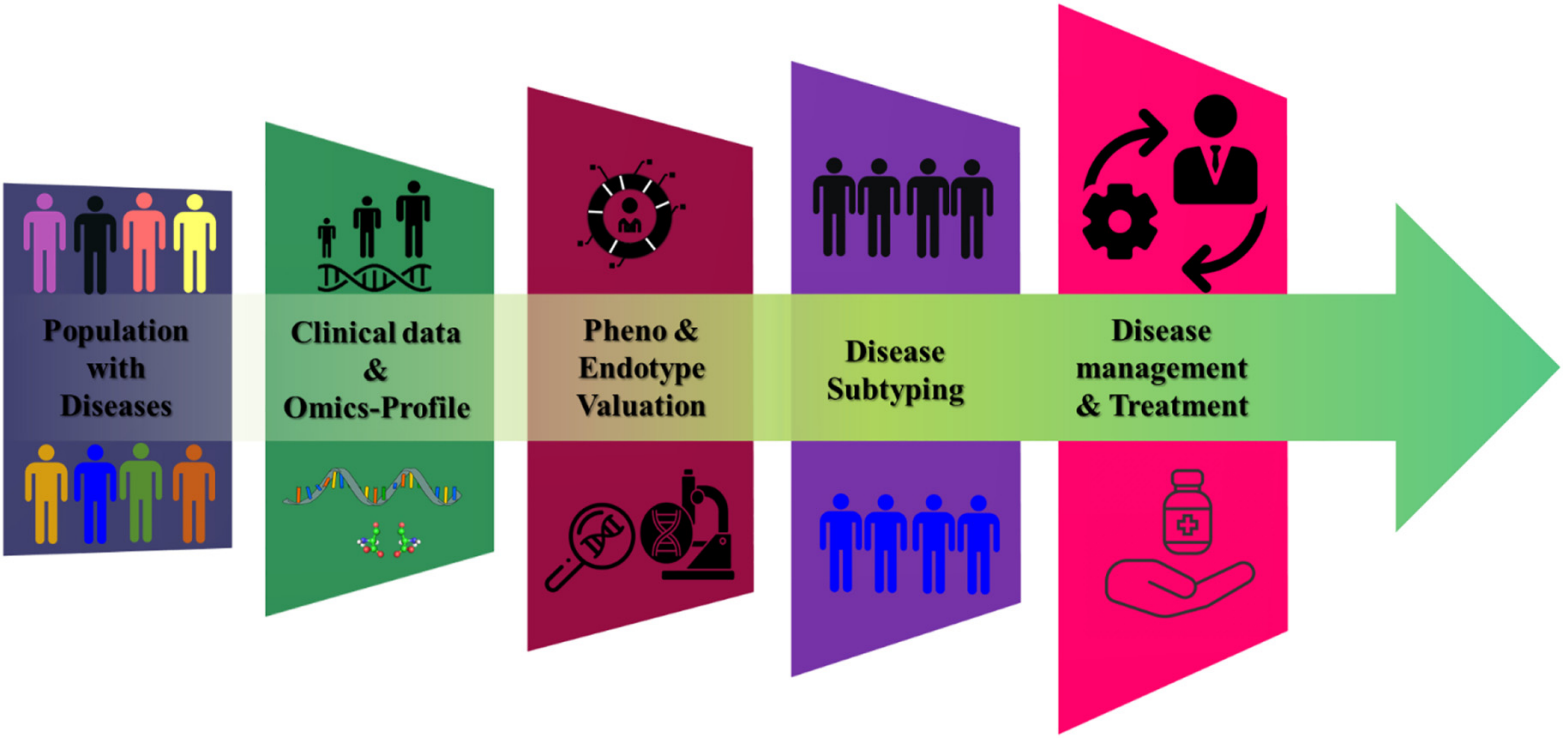
Framework for integration of clinical and multi-omics data for improved disease subtyping within the disease population.

Nevertheless, to bring the concept of exposome to realization, a few factors such as accurate measurement of environmental exposures, biological responses, and the dynamic nature of exposome have to be facilitated [39]. To address these challenges, devices have been developed using high-throughput ‘omics’ (epigenomics, transcriptomics, proteomics, adductomics, and metabolomics) and ‘onics’ (mass-spectrometers, wearable devices, sensors, and NMR) technologies [12, 39], [40], [41], [42], [43] which has in turn dramatically revamped personalized medicine. These technologies enable detailed biological phenotyping (a process of measuring the observables of an organism due to the interaction of its genotype with the exposome) [44, 45]. Digital phenotyping, defined as “moment-by-moment quantification of the individual-level human phenotype *in-situ* using data from smartphones and other personal digital devices” [46], is the state-of-the-art outcome of these constantly evolving technologies [45, 47, 48]. Therefore, we intend to discuss in detail, the ‘omics’ and ‘onics’ technologies that have taken precision medicine [43] to the next level. The Framework for integration of clinical and ‘multi-omics’ data for improved disease subtyping within the disease population is depicted in Figure 2. Even though PoCT is promising in health care, several challenges need to be addressed. The PoCT devices are primarily for one-time use, which results in a higher cost of the device. Additionally, most of the test strips used currently are sensitive to external factors such as light, humidity, and temperature, which makes storing and transporting without contaminating the device more complex. There is also a need for synchronizing the measurements of PoC devices to centralized systems and between different brands of these devices. The ability to simultaneously measure several analytes selectively and sensitively on the same cartridge is also a challenge that needs to be addressed [49].

## 3 ‘Omics’ and ‘onics’

### 3.1 ‘Omics’

We saw that coexistence of ‘-onics’ and ‘-omics’ technologies have changed the personalized medicine landscape [3, 32]. Development in DNA sequencing research has enabled individual genome sequence analysis and access to detailed knowledge in genomic contributions to health and disease which has aided in a more precise approach to patient care [50, 51]. DNA contains information on an individual’s hereditary and ‘biochemical properties of terrestrial life [52, 53]. Therefore, inference and measurement of these sequences are imperative in understanding genomic contributions to health and disease or personal genomics [50–52]. Advanced sequencing technology developed over the past decade allows a detailed understanding of the human exome sequencing (which studies the protein-coding areas, exons, of DNA) and genome sequencing (it analyzes exome as well as non-protein-coding DNA) for precision and personalized therapy [33]. In cancer diagnosis, genome and detailed exome sequencing of cancer cells have helped to identify driver mutations, previously unknown mutational mechanisms (e.g., chromothripsis [54], kataegis [55]), and behavior of various cancer subclones over space and time [33]. Thus, in contrast to genotyping, targeted sequencing (exome constitutes approximately 3% of the genome) allows the sequencing of relevant regions like the whole exome [52, 56]. The advanced ‘omics’ technology and its function are detailed in Table 1.

In addition to these genome studies, exposome knowledge can significantly improve the prediction accuracy in phenotypes [57]. However, the biggest challenge is keeping track of the variability in an individual’s exposome in a lifetime and its dynamic effects. Exposomics, which studies the exposome, mainly analyses internal and external exposure effects of a person’s environment and lifestyle. The internal exposure is assessed using epigenomics, transcriptomics, proteomics, adductomics, and metabonomic. Techniques such as biomarkers, big data, and statistical overview from data mining can help analyze the exposome’s effect on an individual. External exposure assessment can be done using various sensors and survey instruments. Some challenges posed in exposomic measurement are factors like large variety of chemicals and metabolites in the environment, their low abundance, and lack of standard measurements [58]. Developing advanced ‘onics’ devices can address these issues to a greater extent. 

**Table 1:** Omics and its feature in PoC.

S. No.	Omics	Definition
1	Epigenomics	The epigenome holds necessary information to regulate gene expression using methods like DNA methylation and histone modification [59, 60].
2	Transcriptomics	Transcriptome is the measure of abundance and activity of all ribonucleic acids (RNAs) in time and space which helps in understanding the molecular activity in cells that affects human physiology and pathology [61].
3	Proteomics	Proteomics is the study of varieties, roles and dynamics of protein in a cell; sets of all protein isoforms, their modifications and interactions; description of protein and their higher-order complexes, that is, everything post-genomic [61, 62]. The dynamics of a cell is more accurately predicted by proteome and hence it can lead to better biomarkers of disease and prognosis [63].
4	Adductomics	Due to the action of the enzymes that modify DNA or due to exposure to endogenously and exogenously produced electrophiles and oxidants, DNA of living cells undergo structural modifications, creating DNA adducts [64, 65]. The measurement of such DNA adducts provides molecular evidence on the damage occurred to DNA [66, 67].
5	Metabolomics	Metabolomics deals with the systematic identification and quantification of all metabolites in a biological system [68]. Metabolomes are highly linked to various diseases [53]. The advantages of metabolomics over other techniques are that biochemical activities can be directly read from metabolite concentrations and majority of biological processes are based on metabolism [68].

### 3.2 ‘Onics’

The ‘omics’ information combined with ‘onics’ can lead to automation, high precision, and simplification of PoC tools [69]. From biochips to CMOS imagers or ion sensing arrays, the two technologies have delivered results in personalized medicine in ways never envisioned [70]. A few examples are fluorescent dyes used in DNA sequencing; fluorescence technologies (ion channel probes and fluorescent probes) used in drug discovery; cellular biosensors and extrinsic cellular sensors for health monitoring and disease diagnosis; and high-resolution imaging for the analysis of anatomy and internal organs [71, 72].

Combining these two evolving technologies and their link with information technology enables the development of novel decentralized PoCT instruments [1]. Typical PoCT categorizes devices into portable handheld devices (e.g., test strips [73]) and sizeable bench-top ones (NMR spectroscopy [74–77] with complex built-in components [78]. The handheld devices built using micro-fabrication methods work on automated preparation of samples, analysis, assay steps, and signal detection. Bench-top devices are versions of central lab equipment but with reduced complexity and size [79].

Some of the commonly used PoC instruments are mass spectrometers [80, 81], spectroscopes [82, 83], smart wearable devices [84–86], imagers [87, 88] and transcranial electric stimulation (TES) [89, 90]. Moreover, next-generation PoC devices such as paper-based diagnostic tools, novel assay formats, and lab-on-a-chip platforms are imminent [91]. These instruments incorporate many built-in ‘onics’ components. One of the major devices employed in many PoC instruments is biosensors for monitoring analytes (there are three basic types of analytes – proteins, nucleic acids, and small molecules [92]). Biosensors are analytical devices that detect these analytes using the electrochemical method (converts biochemical processes into electric signals) or optical method (uses methods like fluorescence or reflection spectroscopy [93, 94]. Research has advanced that biosensors are used for fetching real-time physiological data via dynamic, non-invasive methods from biofluids such as sweat or tears [95]. Integrating another component, complementary metal-oxide-semiconductor (CMOS), in various sensing elements has enabled the development of CMOS-based sensors for targeted therapies in PoC [96–98]. The inclusion of CMOS has brought many advantages, such as lower power consumption via on-chip temperature regulation, lowered number of interconnects, and less interference from external electromagnetic radiations [98]. Sonication and high-intensity UV lasers have enabled efficient and instantaneous photochemical crosslinking of protein-DNA interactions (method used in histone modification) *in vitro* and *in vivo* [99–102]. Hardware platforms like Field Programmable Gate Array (FPGA) have great potential in rolling out personalized care for large number of patients [103]. The crucial part that decides the efficiency of any such instrument is the material used to build it. The sensitivity, selectivity, absorptivity, durability, and several other properties come to play for any instrument to have its desired function.

## 4 Materials in PoCT

As mentioned before, materials used to build these PoC devices play a vital role in their efficiency. Conventionally, silicon and compound semiconductors are used in making PoC devices. Mirroring resonator devices for integrated lab-on-a-chip systems built using silicon [104]; microfluid-based PoC devices [105, 106] with a wide range of biosensor applications demonstrated using glass, silicon, polymer, and paper are examples of PoC devices built from conventional materials [79]. The Discovery of materials with new dimensionalities and functionalities has been the driving force for all technological progresses [26]. These technologies, especially nanomaterial techniques, have a vital role in developing novel PoC devices that are miniaturized, multiplexed, wireless, and accurate [1, 77]. The responsivity mainly determines the efficiency of the PoC instrument, selectivity, and sensitivity of the material to factors such as pH, light, temperature, magnetic field, analytes, and chemical compounds, and their ability to consequently change their properties in a controlled manner [91, 107]. The use of nanomaterials has helped achieve these milestones to a great extent. Gold [108], magnetite [109], and silver [110] nanoparticles are used for signal enhancement to increase sensitivity in lateral flow immunoassays (LFA). Gold nanorod molecular probes are used in optical biosensors to detect target DNA [94]. Molecularly imprinted polymers are used for realizing biorecognition surfaces in biosensors [111–113]. Magnetic nanoparticles (iron oxide [114–116]) are used for targeted drug delivery. Zinc oxide and titanium dioxide nanoparticles are used for skin protection [117, 118]. Sodium molybdenum bronze nanoparticles have been successfully demonstrated in near infra-red photo-amplified sonodynamic therapy to eliminate staphylococcus aureus bacterial infection [119]. Although a lot has been achieved with these materials, extensive research is still underway to improve the sensitivity and accuracy of PoC devices.

Thus, with the discovery of 2D materials, researchers have been focusing on integrating them into various PoC devices [120–122], the reason being the unique mechanical [123], optical [124], electrical [77, 125], chemical [126, 127], and electrochemical [128, 129] properties of the ultrathin 2D materials and its ability to respond to specific disease models [130]. Their unique properties, such as planar structure; mechanical flexibility; high surface-to-volume ratio; tunable electronic, optical, and electrochemical properties; porosity; sensitivity; selectivity; and fluorescence emittance/quenching, make them more compatible with current fabrication techniques and a good choice for various healthcare applications, especially wearable sensing devices [130–133]. These unique properties result from the confinement of electrons to a layer that alters the electronic, optical, physical, and chemical properties of 2D materials from that of their parent bulk materials [134].

Graphene is the first 2D material discovered and is widely demonstrated in various healthcare devices [135–139]. Graphene has a high surface area (2630 m^2^/g), high electrical conductivity (1000 S/m), thermal conductivity (3000–5000 W/mK), and mechanical strength (Young’s modulus of ∼1.0 TPa), and tunable bandgap [128, 138, 140, 141]. The planar nature, high surface area, and low electronic noise from the thermal effects of graphene enable a more significant number of analyte-surface binding sites and good modulation of electronic properties. This, in turn, improves sensitivity even for low concentrations of analytes [135, 136, 142]. The high conductivity and small graphene bandgap favor electrons conduction from biomolecules [137, 143].

**Figure 3:**
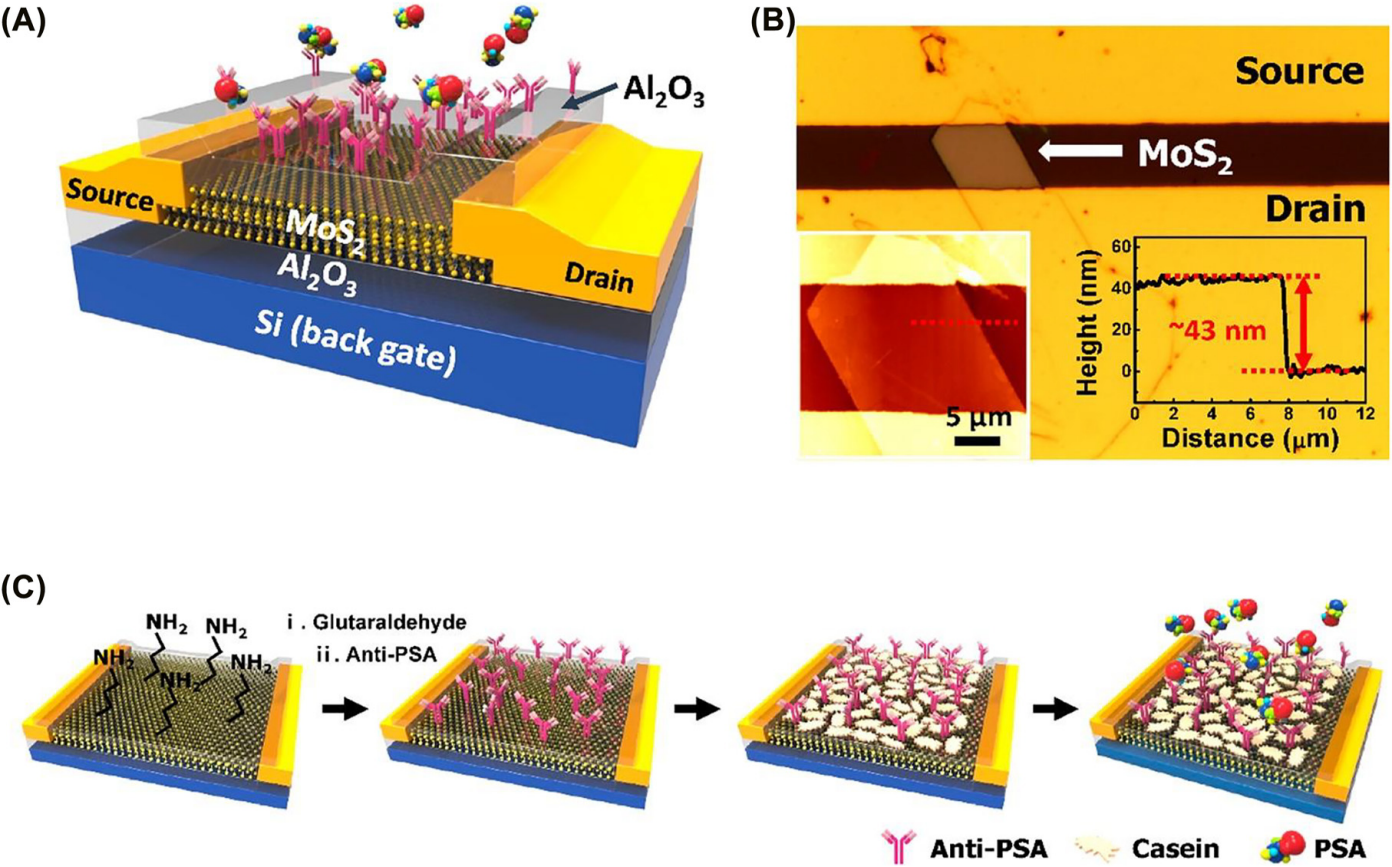
Schematic representation of MoS_2_ based PoC dvices for biological sample analysis. Reproduced with permission from [148] Copyright @The American Chemical Society 2027.

Although, due to the zero bandgap of graphene, it gives a low on/off ratio in FETs, limiting its application in biomedical devices for which semiconducting properties are necessary [130]. Currently, molybdenum-based 2D nanostructures also emerging as exciting materials in the biomedical sector. Specifically, their electronic, chemical, and optical properties make them promising therapeutic agents [144]. For example, applying MoS_2_ nanosheets as effective sonosensitizers for photothermal-enhanced sonodynamic antibacterial therapy proves the prospect of molybdenum-based 2D materials in PoCT [130, 144]. MoS_2_ Nanostructures that possess a 2D nature have been used for biosensing based on the electrochemical phenomenon. There has been extensive exploration of the MoS_2_ sheets in the form of electrode materials in biosensors. MoS_2_ nanosheets display strong fluorescence in the visible range because of their direct bandgap, which makes MoS_2_ a suitable and appropriate candidate for optical biosensors [145]. 1-D MoS_2_ displays good electrical characteristics and is analog to carbon nanotubes (CNTs). A few applications using 2D materials such as MXene-based nanopore for the detection of different types of DNA bases [146]; graphene-hBN heterostructure for DNA sequencing [147]; as shown in Figure 3 [148], MoS_2_ in mass spectrometry for the detection of small molecules [149] are reported on PoC devices using 2D materials. The potential for more 2D materials with better and new functionalities has scope for exploration in PoC medicine [150]. For improvement of current materials, various parameters such as resolution or feasibility of detecting analytes, scalability, compatibility, reproducibility, and sensitivity of nanomaterials are considered. The cost as well as method of operation should also be taken into account.

## 5 Scope of 2D materials based ‘onics’ devices

### 5.1 Photonics

Photonics technology has benefited biomedical sciences immensely over the last few decades. The uses of light in imaging and spectroscopy are popular. Sensor miniaturization enabled advanced imaging technologies and the development of multichannel sensor technologies resulted in novel photonic devices that led to the knowledge of the genetic and molecular bases of various diseases [71]. Some of the imaging technologies in use are magnetic resonance imaging (MRI), computed tomography (CT), nuclear medicine, and optical imaging [71]. These discoveries enabled personalized diagnosis and therapy.

Photodetectors are essential components in many photonic devices used for PoCT. For example, PoCT devices based on fluorescence-labeled immunoassays depend on the sensitivity of photodetectors. The high sensitivity of photodetectors enable fluorescence signal detection even for low concentrations of a microfluidic channel [151]. Another technique, photoplethysmography (PPG), is used to perform *in vivo* measurements of arterial pulsation. It is a real-time, noninvasive analysis obtained from variation in light intensity when interacting with biological systems. The significant components of PPG are irradiating light sources and photodetectors to detect light scattered from biological tissues [152]. Including 2D material-based photodetectors can enhance the performance and miniaturize the device. 2D materials and their heterostructures have exhibited high photo-detecting performance with an external quantum efficiency of 30% (graphene-WS_2_-graphene heterostructure); stable responsivity (55.06 mA W^−1^), and increased sensitivity in visible light and near-infrared range (Bi_2_Te_3_); and high photo gain of around 10^8^ electrons/photons (PbS quantum dot coated graphene) [153–156].

Optical tweezers that work on the principle of mechanical effects of electromagnetic radiation can be manipulated by the analysis of single cells such as mammalian cells, E-coli, red blood cells, nerve cells, and stem cells [157]. The main advantages of optical tweezers are that they use no contact forces to manipulate cells and can be used in a liquid medium. Optical tweezers use a microscopic objective lens and standard Gaussian laser beam [157]. This device can be further improved by using a non-Gaussian laser beam, dual beams, and multiple traps; other techniques like Raman spectroscopy or confocal microscopy. Additionally, optical tweezer are integrated with microfluidic devices for single-cell manipulation [157, 158]. The visual and electronic properties of graphene oxide have been used to build optical tweezers to study E-coli bacteria and can be extended to learning cell metabolism, cytotoxicity, and cell stimuli [159]. Taking advantage of the tunable and nonlinear optical properties of 2D materials, other 2D materials can be studied to enhance the performance of the optical tweezers.

Optical biosensors are essential in PoC as they can be used for various functionalities such as diagnosing multiple diseases like cancer, monitoring cellular activities, and analyzing protein interactions. The introduction of nanotechnology and 2D nanostructures has only resulted in advanced optical biosensors with more accuracy, which is a requirement in PoCT. Surface plasmon resonance is a photonics-based sensor technology that uses the refractive index of the analytes to detect various metabolites [160]. Bio-SPR is an advanced SPR in which the biomolecules such as DNA, RNA, virus, uric acid, protein, glucose, and dopamine binds to the surface of the sensor and thereby causes an increase in the refractive index, which in turn changes the refraction angle of light [161–167]. This shift in the curve is directly proportional to the rise in mass, and the changes are observed as shift in resonance angle of the refracted light [160].

Graphene oxide (GO) contains sp^2^-and sp^3^-hybridized carbon atoms and different oxygen-containing functional groups such as hydroxyl, epoxy, and carboxyl on its basal plane and sheet edges, which can be used for immobilization of bio molecules [168]. In recent years, the functionalized GO has been exploited to fabricate biosensors for detecting various biosamples. Other than SPR, several fiber optics and refractive index-based grating methods such as fiber Bragg gratings (FBGs), long-period gratings (LPG), and tilted fiber gratings (TFTs) are also used for label-free, real-time, multiplex, and in-line determination of biosamples. Xianfeng Chen et al. developed a dual-peak long-period grating (dLPG)-based biosensor with GO fictionalized long-period grating for ultrasensitive label-free detection of Immunoglobulin G (IgG). With GO deposition, the refractive index (RI) sensitivity of dLPG will be enhanced by 200% and 155% in the low RI (1.333–1.347) and high RI (1.430–1.441) regions, respectively. Here, the GO-dLPG will be biofunctionalized with IgG and a quantifiable optical signal will be detected, which corresponds to the analyte’s refractive index change in which the IgG and anti-IgG binding interaction occurrs. The achievable limit of detection (LoD) with GO-coated dLPG is 7 ng/mL, which is 10-fold higher than the noncoated dLPG biosensor and 100-fold more elevated than the LPG-based immunosensor [168].

The exceptional biocompatibility of GO allows surface modification of other biological molecules. For example, staphylococcal protein A (SPA) functionalized on GO for selective detection of IgG and the tilted fiber Bragg grating (TFBG)-based SPR enables LoD of about 0.5 μg/mL. The excellent biocompatibility of SPR and GO, and SPA further amplifies the detection signal and improves the sensor’s sensitivity. It has been reported that the inclusion of 2D materials such as MoS_2_ and graphene in SPR sensors for the detection of different types of cancers such as Jurkat, HeLa, PC12, MDA-MB-231, and MCF7 has resulted in increased biocompatibility and enhanced performance of the device [168].

**Figure 4:**
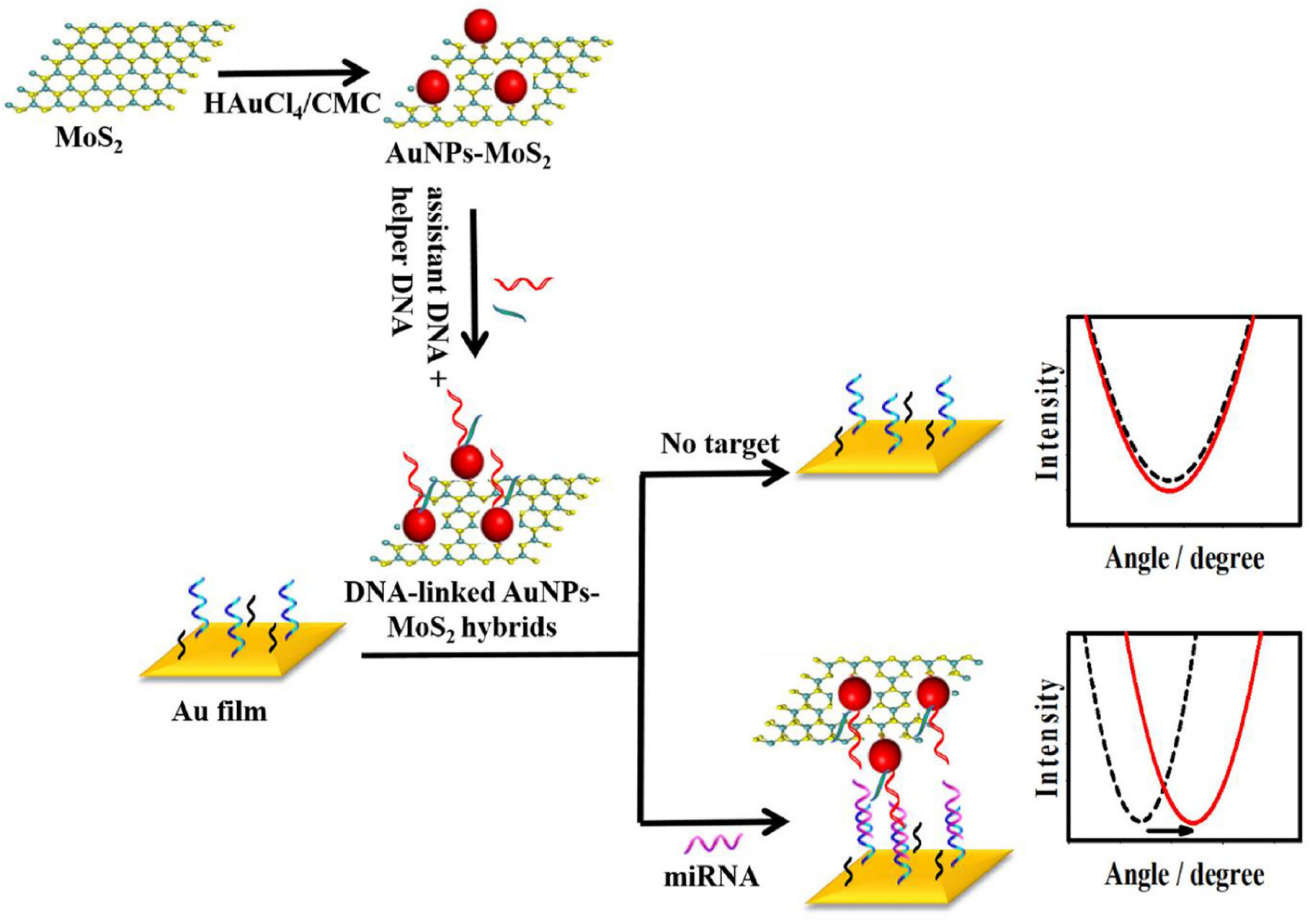
The schematic illustration of the SPR biosensor based on the AuNPs-MoS_2_. Reproduced with permission from [169] Copyright @ Elsevier 2017.

Moreover, results indicate that MoS_2_ performs better in terms of figure of merit (FOM) (6654.54 RIU^−1^) and LoD (0.43 × 10^−5^ RIU) than graphene. The basic principle and mechanism of SPR biosensors based on MoS_2_ is depicted in Figure 4 [169]. Other than MoS_2_ and graphene, other 2D materials such as ZnO and WS_2_ are also used in SPR biosensors to detect biological samples (Tables 2 and 3).

**Table 2:** 2D materials for photonics based omics applications.

S.NO	Nanomaterial	Device	Metabolite	Sensitivity	LoD	Ref
1.	Graphene oxide	SPR- TFBG	Immunoglobulin G (IgG)	0.096 dB/(μg/mL)	0.5 μg/mL	[170]
2.	Graphene oxide	Fiber grating device dLPG	Immunoglobulin G (IgG)	RI (1.430–1.441)	7 ng/mL	[171]
3.	MoS_2_	FO-SPR	Cancer cells	232.33 deg./RIU	0.43 × 10^−5^ RIU	[172]
4.	Graphene	FO-SPR	Cancer cells	231.64 deg./RIU	0.435 × 10^−5^ RIU	[172]
5.	Tin selenide (SnSe)α-SnSe, δ-SnSe, ε-SnSe,	FO-SPR	DNA hybridization	3225 nm/RIU, 3300 nm/RIU, 3475 nm/RIU		[173]
6.	Phosphorene-graphene/TMDC	FO-SPR	DNA hybridization	4050 nm/RIU		[174]
7.	MoS_2_	FO-SPR	Bovine serum albumin	RI: 1.3420	0.29 μg/mL	[165]
8.	ZnO nanorods	FO-LSPR	Prostate-specific antigen	–	0.51 pg/mL	[175]
9.	Ti_3_C_2_ MXene	FO-SPR	Carcinoembryonic antigen	–	0.07 fM	[176]
10.	Phosphorene-WS_2_	SPR	DNA hybridization	187°/RIU		[177]
11.	MoS_2_-GO	FRET	Mycotoxin, aflatoxin B1	–	4.7 pgmL^−1^	[178]
12.	Cu-CdTe	FRET	Mycobacterium tuberculosis IS6110 gene	–	35 pM	[179]
13.	GQDs-MoS_2_	FRET	Epithelial cell adhesion molecule (EpCAM)	–	450 pM	[180]
14.	Graphene oxide	FRET	Botulinum neurotoxin A (BoNT/A)	–	1 fg/mL	[181]
15.	Graphene oxide	FRET based microfluidic chip	Cancer cells, CCRF-CEM cells	–	25 cells mL^−1^	[182]
16.	Graphene oxide and graphene dot	FRET	*Campylobacter jejuni*	–	10 CFU/mL	[183]
17.	Graphene oxide	FRET	Metalloproteinase 2	–	2.5 ng/mL	[184]
18.	GQDs- pyrene	FRET	miRNAs	–	100 pM	[185]
19.	Graphene oxide	FRET	Thrombin	–	2 nM	[186]
20.	Graphene oxide	FRET	Rotavirus	–	105 pfu ml^−1^	[187]
21.	Graphene oxide	FRET	DNA hybridization	–	5 pM	[188]
22.	Graphene oxide	FRET	DNA hybridization	–	75 pM	[189]
23.	Graphene oxide	FRET	*Listeria monocytogenes*	–	100 fg/μL	[190]
24.	Graphene oxide	FRET	DNA	–	40 pM	[191]
25.	Graphene oxide	FRET	*Staphylococcus aureus* DNA	–	6.25 pM	[192]
26.	Graphene quantum dots (GQDs) and carbon nanotubes (CNTs)	FRET	DNA	–	3.6 nM (21 bases)	[193]
27.	Graphene quantum dots	FRET	mecA gene sequence of *Staphylococcus aureus*	–	1 nM	[194]
28.	Graphitic carbon nitride nanosheet	FRET	DNA	–	2.1 nM	[195]
29.	Graphitic carbon nitride nanosheet	FRET	DNA	–	75 pM (15 bases)	[196]
30.	WS_2_	FRET	MicroRNA	–	300 fM	[197]
31.	Graphdiyne/graphene quantum dot	FRET	miRNA-21	–	0.5 pM	[198]

**Table 3:** 2D nanostructures in electronic devices for genomics.

S. No	Nanomaterial	Device	Technique	Metabolite	LOD	Sensitivity	Ref
1.	MoS_2_	Electrochemical	FET	DNA hybridization	10 fM	17 mV/dec	[199]
2.	Graphene	Electrolyte-gated FET	FET	DNA hybridization	25 aM	24 mV/dec	[200]
3.	Graphene	Liquid-gated FET	FET	DNA hybridization	1 pM	–	[201]
4.	Graphene	Liquid-gated FET	FET	DNA hybridization	10 nM–500 nM	–	[202]
5.	Graphene	Multichannel FET	FET	DNA hybridization	10 pM	–	[203]
6.	Graphene	Multichannel FET	FET	DNA hybridization	100 fM	–	[204]
7.	Graphene	Gated FET	FET	DNA single-nucleotide polymorphism	10 pM to 1 nM	–	[205]
8.	MoS_2_	(DNA)Bio-FET	FET	Doxorubicin (anti-cancer drug)	10^−4^ μM to 50 μM	1.7 × 10^3^ A/A	[206]
9.	MoS_2_	Electrolyte-gated FETs	FET	DNA fragments (chromosome 21 or 13)	1 fM	–	[207]
10.	Graphene	Electrolyte gated FET	FET	DNA hybridization	10 aM	26.5 mV/dec	[208]
11.	Graphene	Multi-channel FET	FET	DNA hybridization	10 pM		[203]
12.	RGO	Liquid-gated FET	FET	Peptide nucleic acid (PNA)–DNA hybridization	100 fM	–	[209]
13.	MoS_2_	(DNA)Bio-FET	FET	Prostate-specific antigen, PSA	1 fg/mL	–	[148]
14.	MoS_2_	(anti-PSA) Bio-FET	FET	Prostate-specific antigen, PSA	1 pg/mL	4.3 V/dec	[210]
15.	MoS_2_	(anti-PSA) Bio-FET	FET	Prostate-specific antigen, PSA	3.75 nM	–	[211]
16.	MoS_2_	(anti-PSA) Bio-FET	FET	Prostate-specific antigen, PSA	100 fg/mL	–	[212]
17.	Graphene	(anti-PSA) Bio-FET	FET	Prostate-specific antigen, PSA	100 fg/mL	20 mV/dec	[213]
18.	Graphene	(anti-PSA) Bio-FET	FET	Prostate-specific antigen, PSA	1 nM	–	[214]

In photonic biosensing, fluorescence resonance energy transfer (FRET) is also an attractive and vital technique in detecting molecular interactions and changes in molecular structure [215]. Graphene and graphene-like (2D) nanosheets such as GO and TMDs have been extensively used to design FRET-based biosensors [216–218]. In particular, several research groups have revealed the intrinsic adsorption and fluorescence-quenching capabilities of layered TMD nanosheets toward fluorophore-labeled single-stranded DNA (ssDNA) [219] and aptamers (artificially synthesized short single-stranded oligonucleotide) [220]. It is reported that 2D-MoS_2_ exhibits a remarkable quenching effect compared to GO [221]. However, we believe that this kind of biosensor has an extensive scope for research and development.

Nuclear magnetic resonance (NMR) is a widely applied spectroscopy technique for identifying and quantifying the presence of chemicals in a complex mixture. NMR is employed to analyze metabolomes in bio samples, which is commonly termed quantitative metabolomics or targeted metabolic profiling [41, 222]. The quantification of plenary metabolites in biosamples reflect cellular activity through metabolite alterations and concentrations. This provides a better understanding of cellular processes and functions. However, one of the challenges faced in NMR spectroscope is the long duration of investigation with a reasonably high signal-to-noise ratio due to the low concentration of active nuclei of interest that even the material with a high specific surface area finds difficulty in detecting (∼1000 m^2^/g for mesoporous silica) [223, 224]. To address this problem, a novel nanoparticle-based strategy is implemented. Matrix-assisted laser desorption/ionization time-of-flight mass spectroscopy (MALDI-TOF-MS) has emerged as an essential tool for analyzing and characterizing a wide range of biomolecules such as proteins [225], peptides [226], and nucleic acids [227].

### 5.2 Electronics

Recently, 2D material-based electronic devices such as sensors gained significant interest in detecting metabolites including glucose; lactose; ascorbic acid; adenosine; and some of the inflammation markers such as reactive oxygen species and proteins, nucleic acids, and bacterial cells. For example, electrochemical sensor-based field-effect transistors (FETs) have emerged as reliable detection techniques for amperometric, impedimetric, and potentiometric measurements. Electronic devices enable electrical stimulation of tissues and selective detection of ions, target DNA strands, proteins, and pathogens by measuring changes in the channel resistance [228]. Membrane-based ion-selective electrodes (ISEs) are introduced as particular detection techniques for tiny ionic species. However, their high cost, and low range of LoD need alternatives. An improved option for ISEs ion-sensitive FETs (ISFETs) is introduced for the electrostatic modulation of the surface potential of a channel. Specifically, the LoD is significantly reduced to picomolar range which is mainly achieved due to an increased sensitivity to electrostatic modulation of 2D materials in comparison with conventional materials such as silicon. Moreover, 2D materials’ pliability makes them suitable for creating miniaturized ISFET arrays on flexible substrates for multiplexed monitoring or spatiotemporal mapping [229].

Graphene-based FET (GFET) [230] was developed to effectively sense toxic mercury ions and showed LoD in the range of 0.1 ppb, which is comparatively higher than commercially available ISEs for mercury. Besides graphene, other 2D materials such as MoS_2_, black phosphorus, and h-BN have also been incorporated into ISFET devices [231, 232]. In particular, 2D materials-based label-free electrical detection of biomolecules with a bioelectronic field-effect transistor (bio-FET) also gained significant interest over 1D nanomaterials. This has been achieved due to its superior properties such as higher surface area [233], simple fabrication process [213], reduced noise [234], and increased sensitivity [202]. Lee et al. [199] reported that 2D MoS_2_ semiconductor channel and oxide gate dielectric layer based Bio-FETs are fabricated for detecting target DNA molecules, which showed a low LoD 10 fM, high dynamic range of 10^6^, and increased sensitivity of 17 mV/dec in the shift of Vth. Additionally, this can be operated at a very low voltage with low power consumption and has excellent potential in many applications such as disease diagnostics, environmental monitoring, food safety, and public security based on the detection of DNA molecules.

**Figure 5:**
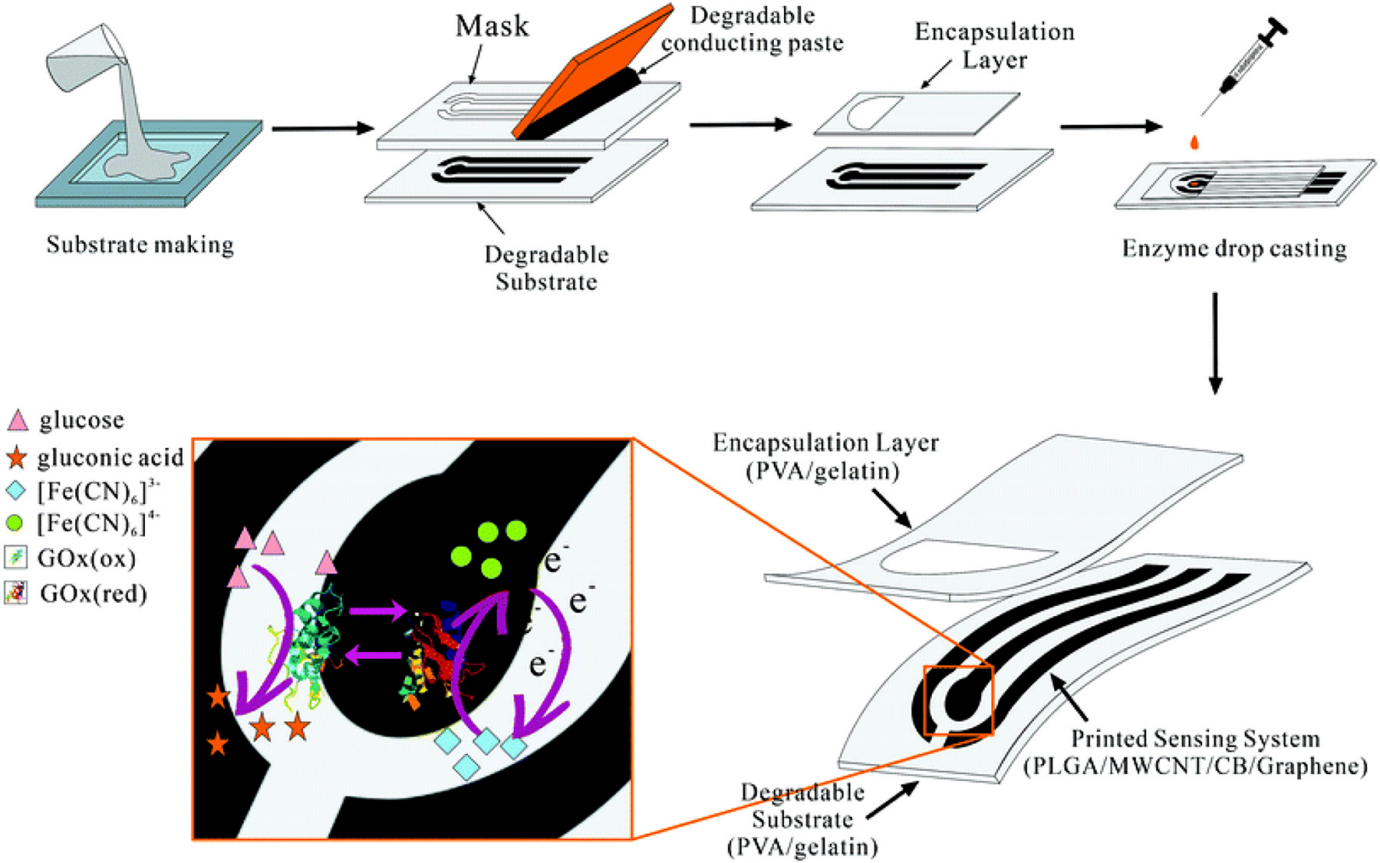
Fabrication process of the fully transient electrochemical strip (up) and the working principle of glucose detection using K_3_Fe(CN)_6_ as an artificial mediator (down). Reproduced with permission from [236] Copyright @ Royal Society of Chemistry 2020.

**Figure 6:**
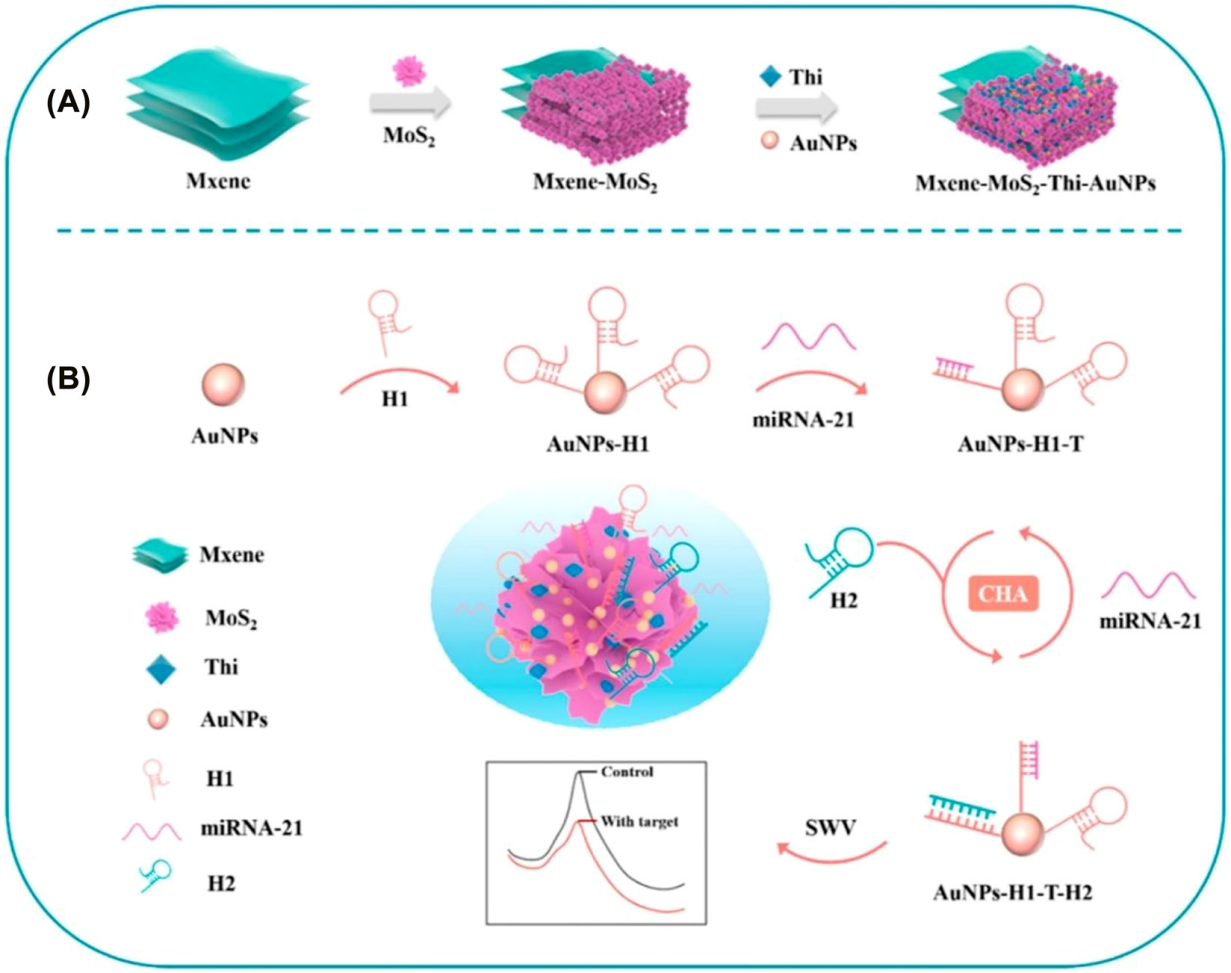
Schematic illustration of the MXene-MoS_2_ nanostructure enhancing electrochemical biosensor for label-free detection of microRNA-21. Reproduced with permission from [238] Copyright @ Elsevier 2022.

TSimple techniques of nanomaterial fabrication has lead to the development of electrochemical sensor strips. Disposable strips is the most common way to monitor biochemical parameters (e.g., glucose, uric acid, cholesterol, etc.) in human blood for noncommunicable disease patients [235]. Ye et al. [236] developed graphene nanosheets, and multiwalled carbon nanotube (MWCNT)-based fully transient electrochemical testing strips for ecofriendly point of care testing of glucose molecules with a sensitivity of 14.33 μA mM^−1^ cm^−2^ (Figure 5). MicroRNAs (miRNAs) are small, noncoding RNA molecules with nearly 18 to 25 nts and are concerned with various cellular activities, including cell proliferation, differentiation, and homeostasis mechanisms. Hence, any deregulation in the function of miRNAs is directly correlated with numerous diseases, including cancer and is regarded as critical biomarkers for cancer diagnosis, therapy, and prognosis. This calls for an urgency in development of a reliable PoC technique for miRNA detection. Thanks to strip-based electrochemical detection sensor in PoC, it facilitates simple, rapid, and reliable detection of microRNAs. Hou et al. [237] developed Ti_3_C_2_T_x_ (MXene)-based test strip with electrochemical disposable DNA circuit to detect miRNAs. This strip allows an LoD of 136 aM (S/N = 3) and dynamic range of 20 fM to 0.4 μM, with a span of 4 orders of magnitude. Notably, they were reported successful in testing eight clinical samples. As depicted in Figure 6, MXene [238] has been combined with MoS_2_ to form a heterostructure to form a label-free electrochemical sensor to detect microRNA-21. This enables a highly folded structure and superior reactive area with an LoD of 26 fM.

Similar to MicroRNAs, DNA biosensors are also used in PoC applications of clinical diagnostics [239], drug interactions [240], and detection [241]. In this regard, commercial screen-printed carbon electrodes (SPCEs) with modified RGO has been realised for detecting DNA hybridization [200], which enabled detection in the low concentration range of 1–200 nM. This suggests that printing of PoC devices with 2D material-based inks on flexible substrates using various printing techniques can take PoCT to next level by allowing miniaturisation, fast manufacturing, and cost-reduction of these devices [242]. However, the key to making these devices commercially viable is ensuring minimal device-to-device variability for a chosen low-cost, high-throughput fabrication method. Additionally, the storage conditions of the sensor, shelf life, and multi-usability need to be methodically studied and optimized to extend their applicability.

## 6 Conclusions

Developments in DNA sequencing resulted in a big leap in understanding health and disease and hence the development of precise approach in personalised patient care. Recently, it has been found that not only personal genomics but also knowledge of an individual’s exposome plays a vital role in the accurate prediction of phenotypes. The advancement in ‘omics’ technology is supported by developments in ‘onics’ technology has fast forwarded the scope of PoCT. A combination of these two technologies has resulted in the development of miniaturized, improved, and novel components such as biochips, CMOS imagers, and devices such as bench-top NMR spectroscopes and smart wearables. However, the inability to build sensitive, affordable, feasible and accurate PoC devices using conventional materials has been the force behind exploring new, more efficient, and cheaper materials. Due to the unique properties of 2Dmaterials, for example, their atomic thickness and large surface-to-volume ratio, they are being extensively explored by researchers in the field of PoCT. These materials own extraordinary optoelectronic properties such as tunability of bandgap, that they have emerged as an important material in photonic applications. This review discusses the relevance and scope of 2D materials in building ‘onic’ and ‘omic’ devices for a highly personalized diagnosis and prognosis. These materials with atomic thickness, biocompatible nature, easy synthesis methods, and unique properties arising from quantum confinement of carriers have the potential to take PoCT to better miniaturization and affordability without compromising on essential characteristics required for the devices such as selectivity, sensitivity, and accuracy.

## Supplementary Material

Supplementary Material Details
