# Supplementary material for: Two-dimensional nanostructures based ‘-onics’ and ‘-omics’ in personalized medicine
Source: Nanophotonics. 2022 Sep 19;11(22):5019–39. doi: 10.1515/nanoph-2022-0439 (PMC11501768; doi:10.1515/nanoph-2022-0439)
Supplement: Supplementary file 1 — Supplementary Material Details [file j_nanoph-2022-0439_suppl.docx]

**Two-dimensional nanostructures based ‘-onics’ and ‘-omics’ in Personalized Medicine**

*Bibi Mary Francis^a#^, Aravindkumar Sundaram^a#^, Rajesh Kumar Manavalan^b^, Weng Kung Peng^d|^, Han Zhang^c*^, Joice Sophia Ponraj^a*^, Sathish Chander Dhanabalan^a*^*

^a^Center for Advanced Materials, Aaivalayam-DIRAC Institute, Coimbatore, Tamil Nadu, India

^b^Institute of Natural Science and Mathematics, Ural Federal University, 620002 Yekaterinburg, Russia

^c^Institute of Microscale Optoelectronics, Collaborative Innovation Centre for Optoelectronic

Science & Technology, Key Laboratory of Optoelectronic Devices and Systems of Ministry of Education and Guangdong Province, College of Physics and Optoelectronic Engineering, Shenzhen Key Laboratory of Micro-Nano Photonic Information Technology, Guangdong Laboratory of Artificial Intelligence and Digital Economy (SZ), Shenzhen University, Shenzhen 518060, P.R. China

^d^Songshan Lake Materials Laboratory, Innovation Park, 523808 Dongguan, China.

^#^Equal Contribution

*Corresponding Author: [joice.ponraj@aaivalayam.com](mailto:joice.ponraj@aaivalayam.com), [hzhang@szu.edu.cn](mailto:hzhang@szu.edu.cn), sathish.dhanabalan@aaivalayam.com

**Abstract**

With the maturing techniques for advanced synthesis and engineering of two-dimensional (2D) materials, its nanocomposites, hybrid nanostructures, alloys, and heterostructures, researchers have been able to create materials with improved as well as novel functionalities. One of the major applications that have been taking advantage of these materials with unique properties is biomedical devices, which currently prefer to be decentralized and highly personalized with good precision. The unique properties of these materials, such as high surface to volume ratio, a large number of active sites, tunable bandgap, nonlinear optical properties, and high carrier mobility is a boon to ‘onics’ (photonics/electronics) and ‘omics’ (genomics/exposomics) technologies for developing personalized, low-cost, feasible, decentralized, and highly accurate medical devices. This review aims to unfold the developments in point-of-care technology, the application of ‘onics’ and ‘omics’ in point-of-care medicine, and the part of two-dimensional materials. We have also discussed the prospects of photonic devices based on 2D materials in personalized medicine and have also briefly discussed electronic devices for the same.

**Keywords:** 2D materials, Point-of-Care technology, Photonics, ‘onics’, ‘omics’

1. **Introduction**

One of the most significant developments in healthcare in the last few decades is the decentralization of diagnosis and prognosis and their personalization to the point of care. This revolution in personalized care is primarily due to the development of a vast number of low-cost, faster, and user-friendly devices [1-3] such as sensors and lab-on-a-chip; genomic technologies such as gene editing [4, 5], 3D genomics [6, 7], functional genomics [8], and epigenomics [9]; and knowledge on exposomics which is a measure of the impact of humans lifetime exposure to lifestyle and environment on their health. These devices and systems are developed by combining knowledge of ‘-omics’ (genomics and exposomics) and ‘-onics’ (electronics and photonics), leading to precise biomarkers of health and disease[10].

Advances in ‘onics’ have led to the miniaturization of devices such as the NMR system with smaller electronic consoles, probes, and microfluidic-based chips, improving its application in point-of-care medical diagnosis [11, 12]. The two layers of ‘omics’ – genomics, and exposomics- have furthered processes such as profiling tumor cells, DNA, and RNA, phenotyping diabetes mellitus, and detecting various pathological states [11]. Additionally, these developments have revolutionized the field of clinical diagnostics with artificial intelligence-based image analysis [13] and the development of wise, connected PoC devices based on the Internet of Things (IoT) [14].

A critical factor in the efficiency of PoC healthcare is the characteristics of the materials used in the devices deployed for diagnosis and prognosis. Here, nanomaterials have an advantage. Compared to conventional silicon-based semiconductor devices, nanomaterials-based devices are compact, low-cost, more sensitive, faster, and lighter [15]. Furthermore, the typical size of nanomaterials matches the size of the components of living organisms, enabling effective interaction between devices and bio systems [2]. Nanomaterials like carbon nanotubes and silicon nanowires have added the advantage of good tunability (from diameter dependence of bandgap) [16]. It has to be emphasized that the distinct advantage of nanomaterials-based devices over conventional biomedical methods is in sensitivity – which means minimal false negatives [2, 17]. However, disparities in dimensions and alignment constrain the use of carbon nanotubes and silicon nanowires in conventional device fabrication. This, along with the need for more sensitivity, accuracy, and reliability, kept pushing researchers to find better alternatives [18, 19]. 2D materials - another class of nanomaterials such as graphene, transition metal dichalcogenides (TMDs), MXenes, and hexagonal boron nitride (hBN) are proving to be more effective in biomedical applications [20].

Advanced synthesis and engineering of 2D materials allow us to create various functionalities via defect engineering, chemical/molecular doping [21-24], and synthesis of heterostructures, nanocomposites, or alloys with other nanomaterials [25-27]. Thus, functionalized 2D materials have a significant role in building in vitro and in vivo diagnostic sensors and imaging devices for protein transducers, drug delivery vehicles, and diagnosis of microbes, cancer cells, etc. [2].

This review discusses the role/scope of 2D materials in ‘onics’ and ‘-omics’-based technologies in personalized medicine. As depicted in **Figure 1**, we start with our view on point-of-care technology (PoCT) - its significance, advantages, developments, and obstacles. We describe ‘-omics’ and ‘-onics’ technologies and the relevance of their union in PoCT. We have also explained the conventional materials used in ‘onics’ technology and their limitations. We clearly demonstrate the complimenting state of 2D materials and the importance of integrating them in PoCT. The scope of 2D materials in ‘onics’ for PoCT is discussed in detail.

1. **Point of care technology (PoCT)**

PoC personalized medicine has the potential to develop highly responsive therapies for various diseases. It considers an individual's unique genetics and exposome and aims at targeted diagnosis, prognosis, and treatment rather than a generalized one [28]. With the support of technology, point-of-care medicine has come a long way with PoC devices that are user-friendly, low-cost, and miniaturized with reduced turnaround time (time between sample collection and analysis) [1]; they enable personalized and decentralized preventive medical screening resulting in patient-specific and timely treatment [1, 29-32]. PoC technology, at present, includes devices ranging from the commonly used blood-glucose testers to viscoelastic coagulation assays [1]. These devices enable constant monitoring of physical conditions (e.g., blood sugar, blood pressure, or stress) and automated data processing, resulting in early detection of diseases. [14].

The advances in genomics have enabled these PoCT since their beginnings in the 1990s [3, 33]. Understanding human genome sequences and developing tools, sophisticated statistics, and computational methods have led to the identification of many human diseases and the realization of genomic medicine [34, 35]. Genomic medicine uses a patient’s genomic information to assess the individual’s or his family’s risk for a particular disease, diagnose rare diseases and improve medicine efficacy [36, 37]. Genomic analysis of cancer has enabled the development of personalized therapeutic agents [38]. A new paradigm, ‘exposome,’ sums up a person’s exposure to micro- (e.g., microbiomes) and macro-environment (e.g., pollution, lifestyle) and complements the concept of the genome. Exposome has a significant impact and greater attributable risk on human health [3, 39]. Environmental factors such as air pollution and characteristics associated with one’s lifestyle can significantly develop various chronic pathologies, including respiratory diseases and diabetes mellitus [39]. This idea that an individual’s environment dramatically influences their traits [37] has overhauled the hypes associated with personalized disease stratification and prevention, which had depended solely on genomic medicine established on the molecular basis of health and disease [3]. This understanding has been advantageous in developing agents that could target patient groups for whom traditional health care has failed [29].

Nevertheless, to bring the concept of exposome to realization, a few factors such as accurate measurement of environmental exposures, biological responses, and the dynamic nature of exposome have to be facilitated [39]. To address these challenges, devices have been developed using high-throughput ‘omics’ (epigenomics, transcriptomics, proteomics, adductomics, and metabolomics) and ‘onics’ (mass-spectrometers, wearable devices, sensors, and NMR) technologies [12, 39-43] which has in turn dramatically revamped personalized medicine. These technologies enable detailed biological phenotyping (a process of measuring the observables of an organism due to the interaction of its genotype with the exposome) [44, 45]. Digital phenotyping, defined as “moment-by-moment quantification of the individual-level human phenotype in-situ using data from smartphones and other personal digital devices” [46], is the state-of-the-art outcome of these constantly evolving technologies [45, 47, 48]. In short, ‘omics’ and ‘onics’ technologies that have taken precision medicine [43] to the next level will be discussed in detail. The Framework for integration of clinical and ‘multi-omics’ data for improved disease subtyping within the disease population is depicted in **Figure 2**. Even though PoCT is promising in health care, several challenges need to be addressed. The PoCT devices are primarily for one-time use, which results in a higher cost of the device. Additionally, most of the test strips used currently are sensitive to external factors such as light, humidity, and temperature, which makes storing and transporting without contaminating the device more complex. There is also a need for synchronizing the measurements of POC devices to centralized systems and between different brands of these devices. The ability to simultaneously measure several analytes selectively and sensitively on the same cartridge is also an obstacle that needs to be addressed [49].

1. **‘Omics’ and ‘Onics****’**
   1. **. ‘Omics’**

We saw that coexistence of ‘-onics’ and ‘-omics’ technologies have changed the personalized medicine landscape [3, 32]. Development in DNA sequencing research has enabled individual genome sequence analysis and access to detailed knowledge in genomic contributions to health and disease for a more precise approach to patient care [50, 51]. DNA contains information on an individual’s hereditary and ‘biochemical properties of terrestrial life [52, 53]. Therefore, inference and measurement of these sequences are imperative in understanding genomic contributions to health and disease or personal genomics [50-52]. Advanced sequencing technology developed over the past decade allows a detailed understanding of the human genome exome sequencing (which studies the protein-coding areas of DNA) and genome sequencing ( it analyzes exome as well as non-protein-coding DNA) for precision and personalized therapy [33]. In cancer diagnosis, exome and genome sequencing of cancer cells have helped to identify driver mutations, previously unknown mutational mechanisms (e.g., chromothripsis [54], kataegis [55]), and behaviour of various cancer subclones over space and time [33]. Thus, in contrast to genotyping, targeted sequencing (exome constitutes approximately 3% of the genome) allows the sequencing of relevant regions like the whole exome [52, 56].

In addition to these genome studies, exposome knowledge can significantly improve the prediction accuracy in phenotypes [57]. However, the biggest challenge is keeping track of the variability in an individual’s exposome in a lifetime and its dynamic effects. Exposomics, which studies the exposome, mainly analyses internal and external exposure effects of a person’s environment and lifestyle. The internal exposure is assessed using epigenomics, transcriptomics, proteomics, adductomics, and metabonomic. Techniques such as biomarkers, big data, and statistical overview from data mining can help analyze the exposome's effect on an individual. External exposure assessment can be done using various sensors and survey instruments. Some challenges posed in exposomic measurement are factors like large variety of chemicals and metabolites in the environment, their low abundance, and lack of standard measurements [58]. Developing advanced ‘onics’ devices can address these issues to a greater extent. Some of the advanced ‘omics’ technology and its function are detailed in **Table 1**.

- 1. **. ‘Onics’**

These ‘omics’ information combined with ‘onics’ can lead to automation, high precision, and simplification of PoC tools [59]. From biochips to CMOS imagers or ion sensing arrays, the two technologies have delivered personalized medicine results in ways never envisioned [60]. A few examples are fluorescent dyes used in DNA sequencing; fluorescence technologies (ion channel probes and fluorescent probes) used in drug discovery; cellular biosensors and extrinsic cellular sensors for health monitoring and disease diagnosis; and high-resolution imaging for the analysis of anatomy and internal organs [61, 62].

Combining these two evolving technologies and their link with information technology enables the development of novel decentralized PoCT instruments [1]. Typical PoCT categorizes devices into portable handheld devices (e.g., test strips [63]) and sizeable bench-top ones (NMR spectroscopy [64-67] with complex built-in components [68]. The handheld devices built using micro-fabrication methods work on automated preparation of samples, analysis, assay steps, and signal detection. Bench-top devices are versions of central lab equipment but with reduced complexity and size [69].

Some of the commonly used PoC instruments are mass spectrometers [70, 71], spectroscopes [72, 73], smart wearable devices [74-76], imagers [77, 78] and transcranial electric stimulation (TES) [79, 80]. Moreover, next-generation PoC devices such as paper-based diagnostic tools, novel assay formats, and lab-on-a-chip platforms are imminent[81]. These instruments incorporate many built-in ‘onics’ components. One of the major devices employed in many PoC instruments is biosensors for monitoring analytes ( there are three basic types of analytes - proteins, nucleic acids, and small molecules [82]). Biosensors are analytical devices that detect these analytes using the electrochemical method (converts biochemical processes into electric signals) or optical method (uses methods like fluorescence or reflection spectroscopy [83, 84]. Research has advanced that biosensors are used for fetching real-time physiological data via dynamic, non-invasive methods from biofluids such as sweat or tears [85]. Integrating another component, complementary metal-oxide-semiconductor (CMOS), in various sensing elements has enabled the development of CMOS-based sensors for targeted therapies in PoC [86-88]. The inclusion of CMOS has brought many advantages, such as lower power consumption via on-chip temperature regulation, lowered number of interconnects, and less interference from external electromagnetic radiations [88]. Sonication and high-intensity UV lasers have enabled efficient and instantaneous photochemical crosslinking of protein-DNA interactions (method used in histone modification) in vitro and in vivo[89-92]. Hardware platforms like Field Programmable Gate Array (FPGA) have great potential in rolling out personalized care for large number of patients [93]. The crucial part that decides the efficiency of any such instrument is the material used to build it. The sensitivity, selectivity, absorptivity, durability, and several other properties come to play for any instrument to have its desired function.

1. **Materials in PoCT**

As mentioned before, materials used to build these PoC devices play a vital role in their efficiency. Conventionally, silicon and compound semiconductors are used in making PoC devices. Mirroring resonator devices for integrated lab-on-a-chip systems built using silicon [94]; microfluid-based PoC devices [95, 96] with a wide range of biosensor applications demonstrated using glass, silicon, polymer, and paper are examples of PoC devices built from conventional materials [69]. The Discovery of materials with new dimensionalities and functionalities has been the driving force for all technological progress [26]. These technologies, especially nanomaterial techniques, have a vital role in developing novel PoC devices that are miniaturized, multiplexed, wireless and accurate [1, 67]. The responsivity mainly determines the efficiency of the PoC instrument, selectivity and sensitivity of the material to factors such as pH, light, temperature, magnetic field, analytes, and chemical compounds, and their ability to consequently change their properties in a controlled manner [81, 97]. The use of nanomaterials has helped achieve these milestones to a great extent. Gold [98], magnetite [99], and silver [100] nanoparticles are used for signal enhancement to increase sensitivity in lateral flow immunoassays (LFA). Gold nanorod molecular probes are used in optical biosensors to detect target DNA [84]. Molecularly imprinted polymers are used for realizing biorecognition surfaces in biosensors [101-103]. Magnetic nanoparticles (iron oxide [104-106]) are used for targeted drug delivery. Zinc oxide and titanium dioxide nanoparticles are used for skin protection [107, 108]. Sodium molybdenum bronze nanoparticles have been successfully demonstrated in near infra-red photo-amplified sonodynamic therapy to eliminate staphylococcus aureus bacterial infection. [109] Although a lot has been achieved with these materials, extensive research is still underway to improve the sensitivity and accuracy of POC devices.

Thus, with the discovery of 2D materials, researchers have been focusing on integrating them into various POC devices [110-112] the reason being the unique mechanical [113], optical [114], electrical [67, 115], and chemical properties [116, 117] electrochemical [118, 119] of the ultrathin 2D materials and its ability to respond to specific disease models [120]. Their unique properties, such as planar structure; mechanical flexibility; high surface-to-volume ratio; tunable electronic, optical, and electrochemical properties; porosity; sensitivity; selectivity; and fluorescence emittance/quenching, make them more compatible with current fabrication techniques and a good choice for various healthcare applications, especially wearable sensing devices [120-123]. These unique properties result from the confinement of electrons to a layer that alters the electronic, optical, physical, and chemical properties of 2D materials from that of their parent bulk materials [124].

Graphene is the first 2D material discovered and is widely demonstrated in various healthcare devices [125-129]. Graphene has a high surface area (2630 m^2^/g), high electrical conductivity (1000 S/m), thermal conductivity (3000–5000 W/mK), and mechanical strength (Young's modulus of ~1.0 TPa), and tunable bandgap [118, 128, 130, 131]. The planar nature, high surface area, and low electronic noise from the thermal effects of graphene enable a more significant number of analyte-surface binding sites and good modulation of electronic properties. This, in turn, improves sensitivity even for low concentrations of analytes [125, 126, 132]. The high conductivity and small graphene bandgap favor electrons conduction from biomolecules [127, 133].

Although, due to the zero bandgap of graphene, it gives a low on/off ratio in FETs, limiting its application in biomedical devices, which needs semiconducting properties [120]. Currently, molybdenum-based 2D nanostructures also emerged as exciting materials in the biomedical sector. Specifically, their electronic, chemical, and optical properties make them promising therapeutic agents [134]. For example, applying MoS_2_ nanosheets as effective sonosensitizers for photothermal-enhanced sonodynamic antibacterial therapy proves the prospect of molybdenum-based 2D materials in PoCT [120, 134]. MoS_2_ Nanostructures that possess a 2D nature have been used for biosensing based on the electrochemical phenomenon. There has been extensive exploration of the MoS_2_ sheets in the form of electrode materials in biosensors. MoS_2_ nanosheets display strong fluorescence in the visible range because of their direct bandgap, which makes MoS_2_ a suitable and appropriate candidate for optical biosensors [135]. 1-D MoS_2_ displays good electrical characteristics and is analog to carbon nanotubes (CNTs). A few applications using 2D materials such as MXene-based nanopore for the detection of different types of DNA bases [136, 137]; graphene-hBN heterostructure for DNA sequencing [138]; as shown in **Figure 3** [139], MoS_2_ in mass spectrometry for the detection of small molecules[140] are reported on POC devices using 2D materials. The potential for more 2D materials with better and new functionalities has scope for exploration in POC medicine [141]. For improvement of current materials, various parameters such as resolution or feasibility of detecting analytes, scalability, compatibility, reproducibility and sensitivity of nanomaterials are considered. The cost as well as method of operation should also be taken into account.

### **Scope of 2D materials based ‘Onic****s’ Devices**

### **5.1. Photonics**

Photonics technology has benefited biomedical sciences immensely over the last few decades. The uses of light in imaging and spectroscopy are popular. Sensor miniaturization enabled advanced imaging technologies, and the development of multichannel sensor technologies resulted in novel photonic devices that led to the knowledge of the genetic and molecular bases of various diseases [61]. Some of the imaging technologies in use are magnetic resonance imaging (MRI), computed tomography (CT), nuclear medicine, and optical imaging [61]. These discoveries enabled personalized diagnosis and therapy.

Photodetectors are essential components in many photonic devices used for PoCT. For example, PoCT devices based on fluorescence-labeled immunoassays depend on the sensitivity of photodetectors. The high sensitivity of photodetectors enables fluorescence signal detection even for low concentrations of a microfluidic channel [142]. Another technique, photoplethysmography (PPG), is used to perform in vivo measurements of arterial pulsation. It is a real-time, non-invasive analysis obtained from variation in light intensity when interacting with biological systems. The significant components of PPG are irradiating light sources and photodetectors to detect light scattered from biological tissues [143]. Including 2D material-based photodetectors can enhance the performance and miniaturize the device. 2D materials and their heterostructures have exhibited high photo-detecting performance with an external quantum efficiency of 30% (graphene-WS_2_-graphene heterostructure); stable responsivity (55.06 mA W^−1^), and increased sensitivity in visible light and near-infrared range (Bi_2_Te_3_); and high photo gain of around 10^8^ electrons/photons (PbS quantum dot coated graphene) [144-147].

Optical tweezers that work on the principle of mechanical effects of electromagnetic radiation can be manipulated by manipulation of single cells such as mammalian cells, E-coli, red blood cells, nerve cells, and stem cells can be achieved by optical tweezers which work on the principle of mechanical effects of electromagnetic radiation [148]. The main advantages of optical tweezers are that they use no contact force to manipulate cells and can be used in a liquid medium environment. Optical tweezers use a microscopic objective lens and standard Gaussian laser beam [148]. This device can be further improved by using a non-gaussian laser beam, dual beams, and multiple traps; other techniques like Raman spectroscopy or confocal microscopy; and optical tweezer are integrated with microfluidic devices for single-cell manipulation [148, 149]. The visual and electronic properties of graphene oxide have been used to build optical tweezers to study E-coli bacteria and can be extended to learning cell metabolism, cytotoxicity, and cell stimuli [150]. Taking advantage of the tunable and nonlinear optical properties of 2D materials, more 2D materials can be studied to enhance optical tweezers' performance.

Optical biosensors are essential in PoC as they can be used for various functionalities such as diagnosing multiple diseases like cancer, monitoring cellular activities, and analyzing protein interactions. The introduction of nanotechnology and 2D nanostructures has only resulted in advanced optical biosensors with more accuracy, which is a requirement in PoCT. Surface plasmon resonance is a photonics-based sensor technology that uses the analyte's refractive index to detect various metabolites [151]. Bio-SPR is an advanced SPR in which the biomolecules such as DNA, RNA, virus, uric acid, protein, glucose, and dopamine binds to the surface of the sensor and thereby causing an increase in the refractive index which in turn changes the refraction angle of light [152-158]. This shift in the curve is directly proportional to the rise in mass, and the changes are observed as the shift in resonance angle of the refracted light [151].

Graphene oxide (GO) contains sp^2^- and sp^3^- hybridized carbon atoms and different oxygen-containing functional groups such as hydroxyl, epoxy, and carboxyl on its basal plane and sheet edges, which can be used for immobilization of bio molecules [159]. In recent years, the functionalized GO has been exploited to fabricate biosensors for detecting various biosamples. Other than SPR, several fiber optics and refractive index-based grating methods such as fiber Bragg gratings (FBGs), long-period gratings (LPG), and tilted fiber gratings (TFTs) are also used for label-free, real-time, multiplex, and in-line determination of biosamples. Xianfeng Chen et al. developed a dual-peak long-period grating (dLPG)-based biosensor with GO fictionalized long-period grating for ultrasensitive label-free detection of Immunoglobulin G (IgG). With GO deposition, the refractive index (RI) sensitivity of dLPG will be enhanced by 200% and 155% in the low RI (1.333–1.347) and high RI (1.430–1.441) regions, respectively. Here, the GO-dLPG will be biofunctionalized with IgG. A quantifiable optical signal will be detected, which corresponds to the analyte's refractive index change in which the IgG and anti-IgG binding interaction occurred. The achievable limit of detection (LoD) with GO-coated dLPG is 7 ng/mL, which is 10-fold higher than the non-coated dLPG biosensor and 100-fold more elevated than the LPG-based immunosensor [159].

The exceptional biocompatibility of GO allows surface modification of other biological molecules. For example, staphylococcal protein A (SPA) functionalized on GO for selective detection of IgG, and the Titled Fiber Bragg Grating (TFBG)-based SPR enables LoD of about 0.5 μg /mL. The excellent biocompatibility of SPR and GO, and SPA joint action further amplifies the detection signal and improves the sensor's sensitivity. It has been reported that the inclusion of 2D materials such as MoS_2_ and graphene in SPR sensors for the detection of different types of cancers such as Jurkat, HeLa, PC12, MDA-MB-231, and MCF7 has resulted in increased biocompatibility and enhanced performance [159].

Moreover, results indicate that MoS_2_ performs better results in terms of the figure of merit (FOM) (6654.54 RIU^−1^) and loD (0.43×10^−5^ RIU) than graphene. The basic principle and mechanism of SPR biosensors based on MoS_2_ is depicted in **Figure 4** [160]. Other than MoS_2_ and graphene, other 2D materials such as ZnO and WS_2_ are also used in SPR biosensors to detect biological samples (**Table 2**).

In photonic biosensing, fluorescence resonance energy transfer (FRET) is also an attractive and vital technique in detecting molecular interactions and changes in molecular structure [161]. Graphene and graphene-like (2D) nanosheets such as GO and TMDs have been extensively used to design FRET-based biosensors [162-164]. In particular, several research groups have revealed the intrinsic adsorption and fluorescence-quenching capabilities of layered TMD nanosheets toward fluorophore-labeled single-stranded DNA (ssDNA) [165] and aptamers (artificially synthesized short single-stranded oligonucleotide) [166]. It is reported that 2D-MoS_2_ exhibited a remarkable quenching effect compared to GO [167]. However, we believe that this kind of biosensor still has an extensive research scope.

Nuclear magnetic resonance (NMR) is a widely applied spectroscopy technique for identifying and quantifying the presence of chemicals in a complex mixture. NMR is employed to analyze metabolomes in bio samples, which is commonly termed quantitative metabolomics or targeted metabolic profiling [41, 168] The quantification of plenary metabolites in biosamples reflects cellular activity through metabolites alterations and concentrations. This provides a better understanding of cellular processes and functions. However, one of the challenges faced in NMR spectroscope is the long duration of investigation with a reasonable signal-to-noise ratio due to the low concentration of active nuclei of interest that even the material with a high specific surface area finds difficulty in detecting (~1000 m^2^/g for mesoporous silica) [169, 170]. To address this problem, a novel nanoparticle-based strategy is implemented. Matrix-assisted laser desorption/ionization time-of-flight mass spectroscopy (MALDI-TOF-MS) is an essential tool for analyzing and characterizing a wide range of biomolecules as proteins [171], peptides [172], and nucleic acids [173].

**5.2. Electronics**

Recently, 2D material-based electronic devices such as sensors gained significant interest in detecting metabolites including glucose, lactose, ascorbic acid, adenosine, and some of the inflammation markers such as reactive oxygen species and proteins, nucleic acids, and bacterial cells. For example, electrochemical sensor-based field-effect transistors (FETs) have emerged as reliable detection techniques for amperometric, impedimetric, and potentiometric measurements. Electronic devices enable electrical stimulation of tissues and selective detection of ions, target DNA strands, proteins, and pathogens by measuring changes in the channel resistance [174]. Regarding membrane-based ion-selective electrodes (ISEs) are introduced as particular detection techniques for tiny ionic species, but later on, their high expenses and low detection limit (LoD) range needs alternatives. An improved option for ISEs ion-sensitive FETs (ISFETs) is introduced for the electrostatic modulation of the surface potential of a channel. Specifically, an LoD is significantly reduced (even down to picomolar) thanks to an increased sensitivity to electrostatic modulation compared to conventional materials (such as silicon). Moreover, 2D materials' pliability makes them suitable for creating miniaturized ISFET arrays on flexible substrates for multiplexed monitoring or spatiotemporal mapping [175].

Graphene-based FET (GFET) [176] was developed to effectively sense toxic mercury ions and showed an LoD of 0.1 ppb, which is comparatively higher than commercially available ISEs for mercury [177]. Besides graphene, other 2D materials have also been incorporated into ISFET devices, such as MoS_2_, black phosphorus, and h-BN [178, 179]. In particular, 2D materials-based Label-free electrical detection of biomolecules with a bioelectronic field-effect transistor (bio-FET) also gained significant interest over 1D nanomaterials. Owing to its superior properties such as higher surface area [180], simple fabrication process [181], reduced noise [182] and increased sensitivity [183]. Lee et al. [184] reported that 2D MoS_2_ semiconductor channel and an oxide gate dielectric layer based Bio-FETs are fabricated against detecting target DNA molecules, which showed a low detection limit of 10 fM, high dynamic range of 10^6^, and increased sensitivity of 17 mV/dec in the shift of V^th^. Additionally, this can be operated at a very low voltage with low power consumption and has excellent potential in many applications such as disease diagnostics, environmental monitoring, food safety, and public security based on the detection of DNA molecules.

Thanks to nanotechnology, the easy nanomaterial fabrication process leads to the development of electrochemical sensor strips. POCT using disposable strips is the most common way to monitor biochemical parameters (e.g., glucose, uric acid, cholesterol, etc.) in human blood for NCD patients [185]. Ye et al. [186] developed graphene nanosheets, and multi-walled carbon nanotube (MWCNT) based fully transient electrochemical testing strips for eco-friendly point of care testing of glucose molecules with a sensitivity of 14.33 μA mM^−1^ cm^−2^  (**Figure 5**). MicroRNAs (miRNAs) are small, non-coding RNA molecules with nearly 18 to 25 nts and are concerned with various cellular activities, including cell proliferation, differentiation, and homeostasis mechanisms. Hence, any deregulation in miRNAs function is directly correlated with numerous diseases, including cancer, regarded as critical biomarkers for cancer diagnosis, therapy, and prognosis, enabling an urgent need to develop a reliable POC technique for miRNA detection. Thanks to strip-based electrochemical detection sensor in POC allows easy, rapid, and reliable detection of microRNAs. Hou et al. [187] developed Ti_3_C_2_Tx (MXene) based on a test strip electrochemical disposable DNA circuit to detect miRNAs. This strip allows the detection limit of 136 aM (S/N = 3) and dynamic range (20 fM to 0.4 μM), with a span of 4 orders of magnitude. Notably, they were practical in tested for eight clinical samples. MXene [188] has been combined with MoS_2_ to form a heterostructure label-free electrochemical sensor to detect microRNA-21. This enables a highly fold structure and superior reactive area through collision with a low detection limit (26 fM).

Similar to MicroRNAs, DNA biosensors are also used in POC applications of clinical diagnostics [189], drug interactions [190], and detection [191]. In this regard, Commercial screen-printed carbon electrodes (SPCEs) were modified RGO for detecting DNA hybridization [192], enabling detection in the low concentration range of 1–200 nM. The key to making these devices commercially viable is ensuring minimal device-to-device variability for a chosen low-cost, high-throughput fabrication method. Additionally, the sensors' storage conditions, shelf life, and multi-usability need to be methodically studied and optimized to extend their applicability.

1. **Conclusion**

Developments in DNA sequencing resulted in a big leap in understanding health and disease and hence the development of precise approaches in patient care. Recently, it has been found that not only personal genomics but also knowledge of an individual’s exposome plays a vital role in the accurate prediction of phenotypes. The advancement in ‘omics’ technology is supported by developments in ‘onics’ technology. A combination of these two technologies has resulted in the development of miniaturized, improved, and novel components such as biochips, CMOS imagers, and devices such as bench-top NMR spectroscopes and smart wearables. However, the inability to build sensitive, affordable, feasible and accurate PoC devices using conventional materials has been the force behind exploring new, more efficient, and cheaper materials. Due to the unique properties of 2Dmaterials, for example, their atomic thickness and large surface-to-volume ratio, they are being extensively explored by researchers in this field. These materials own extraordinary optoelectronic properties such as tunability of bandgap that they have emerged as an important material in photonic applications. This review discusses the relevance and scope of 2Dmaterials in building ‘onic’ and ‘omic’ devices for a highly personalized diagnosis and prognosis. These materials with atomic thickness, biocompatible nature, easy synthesis methods, and unique properties arising from quantum confinement of carriers have the potential to take PoCT to better miniaturization and affordability without compromising on essential characteristics required for the devices such as selectivity, sensitivity, and accuracy.

**Acknowledgment:**

The authors extend sincere thanks to the funding and support from Aaivalayam, India. The research was partially supported by the funding from the Science and Technology Development Fund (Nos. 007/2017/A1 and 132/2017/A3), Macao Special Administration Region (SAR), China, and National Natural Science Fund (Grant Nos. 61875138, 61435010, and 6181101252), and Science and Technology Innovation Commission of the ShenzhenKQTD2015032416270385，JCYJ20150625103619275, and JCYJ20170811093453105). Authors acknowledges the support from Instrumental Analysis Centre of Shenzhen University (Xili Campus). One of the authors Manavalan Rajesh Kumar convey his thanks to the contract no. 40/is2.

**TOC Figure**


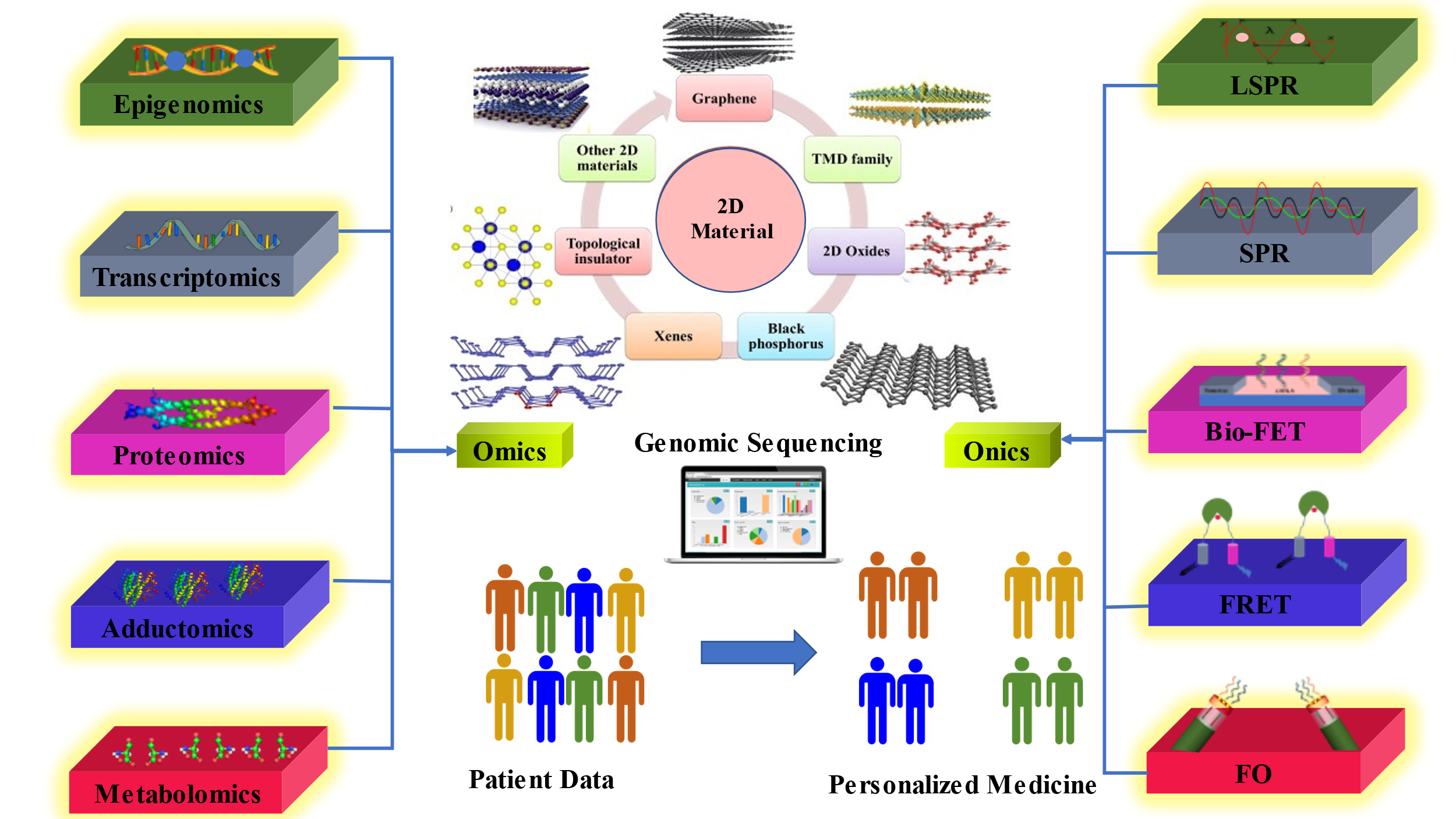


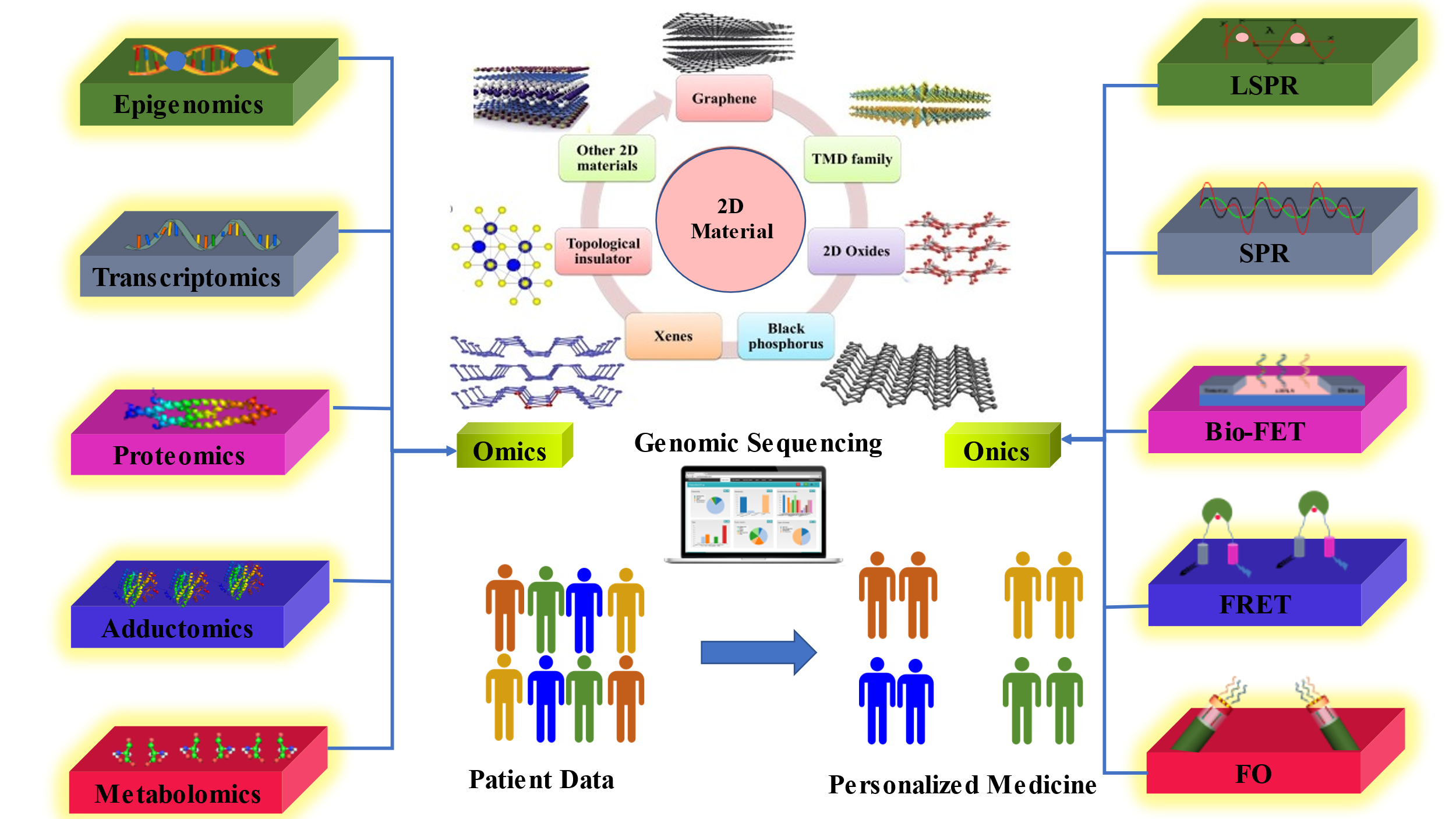


Figure 1: Schematic representation of review oraganization


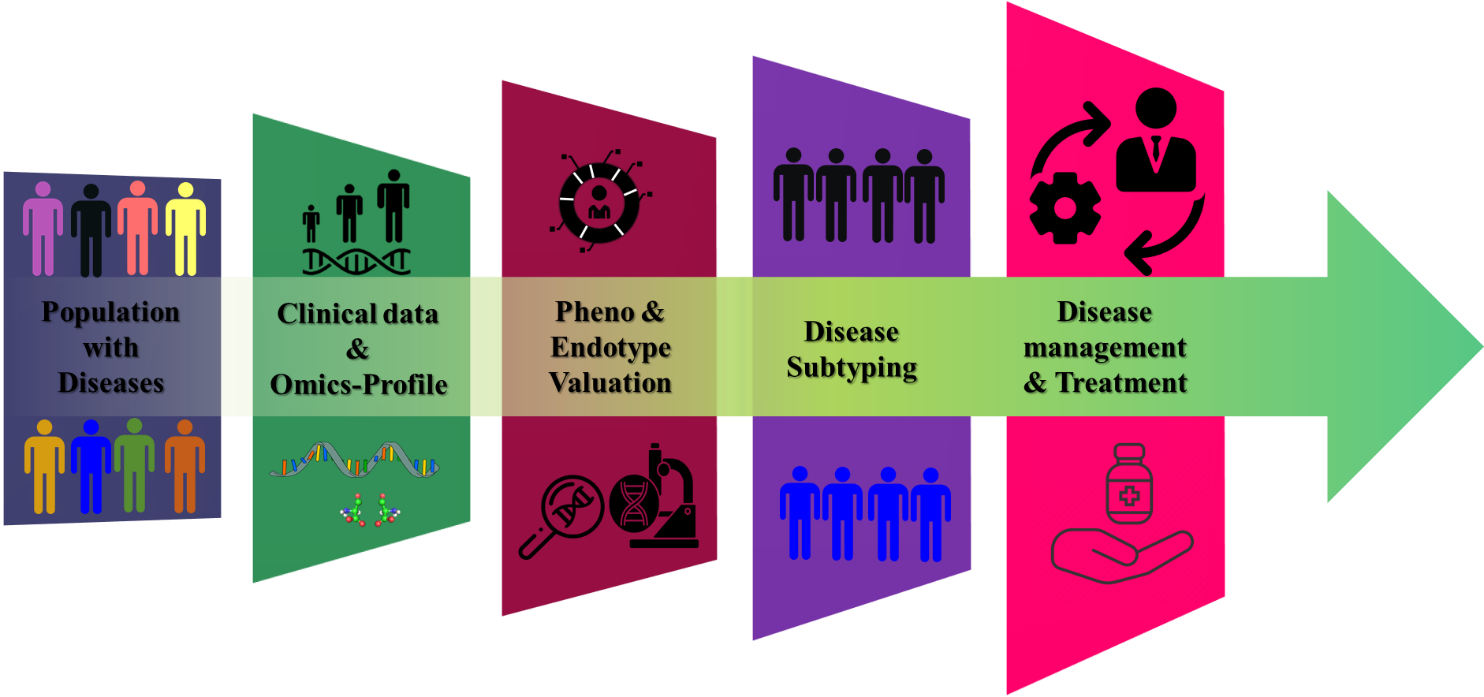


Figure 2: Framework for integration of clinical and multi-omics data for improved disease subtyping within the disease population.


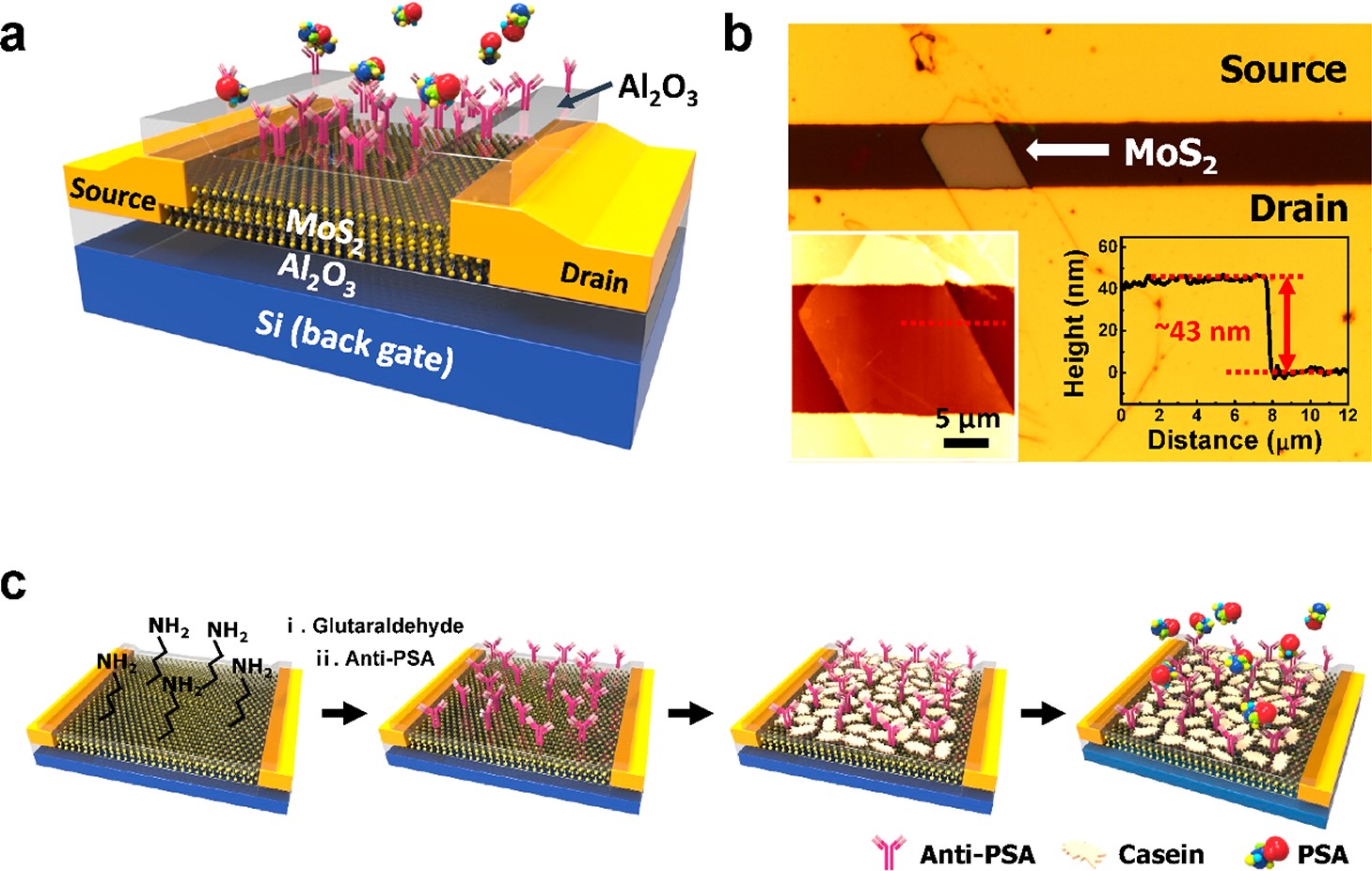


Figure 3: Schematic representation of MoS_2_ based PoC dvices for biological sample analysis. Reproduced with permission from [139] Copyright @The American Chemical Society 2027.


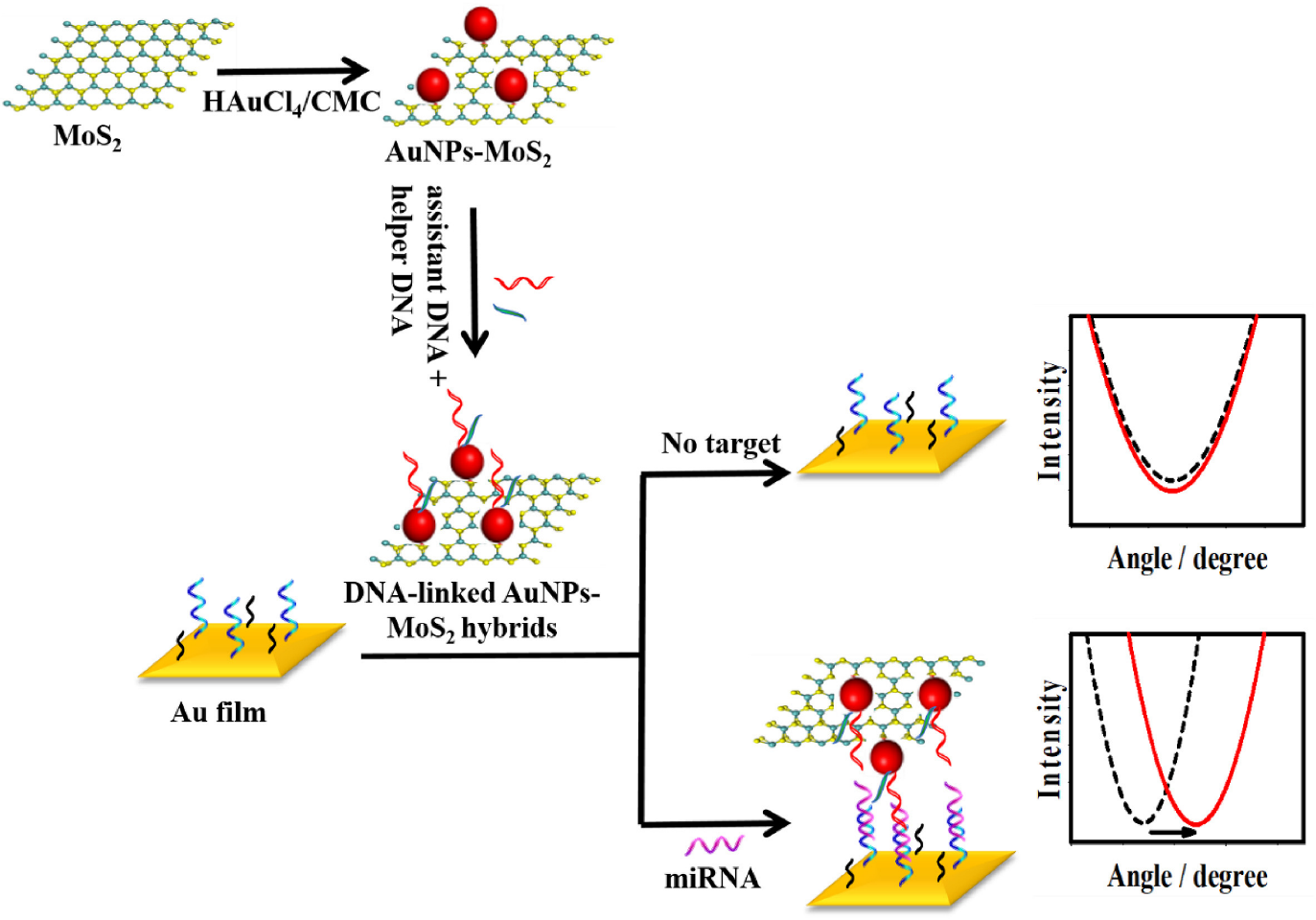


Figure 4:  The schematic illustration of the SPR biosensor based on the AuNPs-MoS_2_. Reproduced with permission from [160] Copyright @ Elsevier 2017.


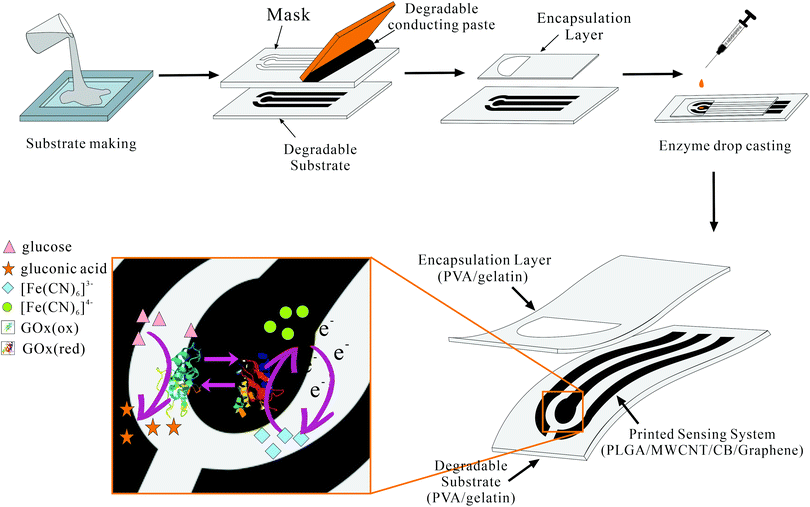


Figure 5: Fabrication process of the fully transient electrochemical strip (up) and the working principle of glucose detection using K_3_Fe(CN)_6_ as an artificial mediator (down).Reproduced with permission from [186] Copyright @ Royal Society of Chemistry 2020.


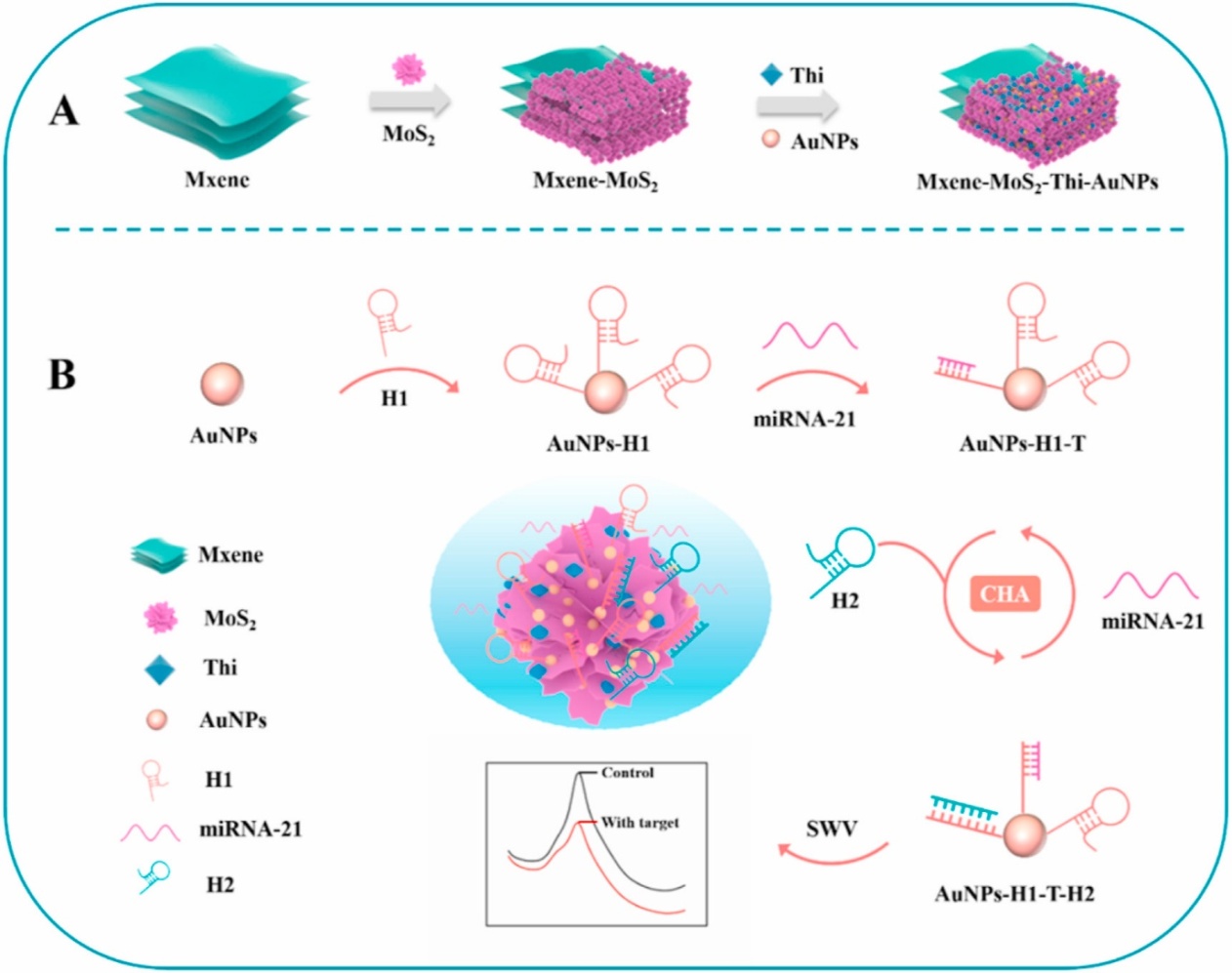


Figure 6: Schematic illustration of the MXene-MoS_2_ nanostructure enhancing electrochemical biosensor for label-free detection of microRNA-21. Reproduced with permission from [188] Copyright @ Elsevier 2022.

| **Table 1. Omics and its feature in PoC** | | |
| --- | --- | --- |
| **S. No.** | **Omics** | **Definition** |
| 1 | Epigenomics | The epigenome holds necessary information to regulate gene expression using methods like DNA methylation and histone modification [193, 194]. |
| 2 | Transcriptomics | Transcriptome is the measure of abundance and activity of all ribonucleic acids (RNAs) in time and space which helps in understanding the molecular activity in cells that affects human physiology and pathology [195] |
| 3 | Proteomics | Proteomics is the study of varieties, roles and dynamics of protein in a cell; sets of all protein isoforms, their modifications and interactions; description of protein and their higher-order complexes, that is, everything post-genomic [195, 196]. The dynamics of a cell is more accurately predicted by proteome and hence it can lead to better biomarkers of disease and prognosis [197]. |
| 4 | Adductomics | Due to the action of the enzymes that modify DNA or due to exposure to endogenously and exogenously produced electrophiles and oxidants, DNA of living cells undergo structural modifications, creating DNA adducts [198, 199]. The measurement of such DNA adducts provides molecular evidence on the damage occurred to DNA [200, 201]. |
| 5 | Metabolomics | Metabolomics deals with the systematic identification and quantification of all metabolites in a biological system [202]. Metabolomes are highly linked to various diseases [53]. The advantages of metabolomics over other techniques are that biochemical activities can be directly read from metabolite concentrations and majority of biological processes are based on metabolism [202]. |

| **Table 2. 2D materials for photonics based omics applications** | | | | | | | | | | |  |
| --- | --- | --- | --- | --- | --- | --- | --- | --- | --- | --- | --- |
| **S.NO** | **Nanomaterial** | | **Device** | | **Metabolite** | | **Sensitivity** | | **LoD** | **Ref** |  |
|  | Graphene Oxide | | SPR- TFBG | | Immunoglobulin G (IgG) | | 0.096 dB/(μg /mL) | | 0.5 μg /mL. | [203] |  |
|  | Graphene oxide | | Fiber grating device  dLPG | | Immunoglobulin G (IgG) | | RI (1.430–1.441) | | 7 ng/mL. | [204] |  |
|  | MoS_2_ | | FO-SPR | | Cancer cells | | 232.33 deg./RIU. | | 0.43×10−5 RIU | [205] |  |
|  | Graphene | | FO-SPR | | Cancer cells | | 231.64 deg./RIU | | 0.435×10−5 RIU | [205] |  |
|  | Tin Selenide (SnSe)α-SnSe, δ-SnSe, ε-SnSe, | | FO-SPR | | DNA hybridization. | | 3225 nm/RIU, 3300 nm/RIU,  3475 nm/RIU | |  | [206] |  |
|  | Phosphorene-graphene/TMDC | | FO-SPR | | DNA hybridization. | | 4050 nm/RIU | |  | [207] |  |
|  | MoS_2_ | | FO- SPR | | Bovine serum albumin | | RI: 1.3420 | | 0.29 µg/mL | [156] |  |
|  | ZnO nanorods | | FO-LSPR) | | Prostate-specific antigen | |  | | 0.51 pg/ml | [208] |  |
|  | Ti_3_C_2_MXene | | FO-SPR | | Carcinoembryonic antigen | |  | | 0.07 fM | [209] |  |
|  | Phosphorene-WS_2_ | | SPR | | DNA Hybridization | | 187°/RIU | |  | [210] |  |
|  | MoS_2_-GO | | FRET | | mycotoxin, aflatoxin B1 | |  | | 4.7 pgmL−1 | [211] |  |
|  | Cu-CdTe | | FRET | | Mycobacterium Tuberculosis IS6110 gene | |  | | 35 pM | [212] |  |
|  | GQDs-MoS_2_ | | FRET | | Epithelial cell adhesion molecule (EpCAM) | |  | | 450 pM | [213] |  |
|  | Graphene oxide | | FRET | | Botulinum neurotoxin A (BoNT/A) | |  | | 1 fg/mL | [214] |  |
|  | Graphene oxide | | FRET based Microfluidic chip | | Cancer cells, CCRF-CEM cells | |  | | 25 cells mL^−1^ | [215] |  |
|  | Graphene oxide and graphene dot | | FRET | | Campylobacter jejuni | |  | | 10 CFU/ml | [216] |  |
|  | Graphene oxide | | FRET | | Metalloproteinase 2 | |  | | 2.5 ng/mL | [217] |  |
|  | GQDs- Pyrene | | FRET | | miRNAs | |  | | 100 pM | [218] |  |
|  | Graphene oxide | | FRET | | Thrombin | |  | | 2 nM | [219] |  |
|  | Graphene oxide | | FRET | | Rotavirus | |  | | 10^5^ pfu ml^−1^ | [220] |  |
|  | Graphene oxide | | FRET | | DNA hybridization | |  | | 5 pM | [221] |  |
|  | Graphene oxide | | FRET | | DNA hybridization | |  | | 75 pM | [222] |  |
|  | Graphene oxide | | FRET | | Listeria monocytogenes | |  | | 100 fg/μL | [223] |  |
|  | Graphene oxide | | FRET | | DNA | |  | | 40 pM | [224] |  |
|  | Graphene oxide | | FRET | | Staphylococcus aureus DNA | |  | | 6.25 pM | [225] |  |
|  | Graphene quantum dots (GQDs) and carbon nanotubes (CNTs) | | FRET | | DNA | |  | | 3.6 nM (21 bases) | [226] |  |
|  | Graphene quantum dots | | FRET | | mecA gene sequence of Staphylococcus aureus | |  | | 1 nM | [227] |  |
|  | Graphitic carbon nitride nanosheet | | FRET | | DNA | |  | | 2.1 nM | [228] |  |
|  | Graphitic carbon nitride nanosheet | | FRET | | DNA | |  | | 75 pM (15 bases) | [229] |  |
|  | WS_2_ | | FRET | | MicroRNA | |  | | 300 fM | [230] |  |
|  | Graphdiyne/graphene quantum dot | | FRET | | miRNA-21 | |  | | 0.5 pM. | [231] |  |
| **Table 3. 2D nanostructures in electronic devices for genomics** | | | | | | | | | | | |
| **S.No** | **Nanomaterial** | **Device** | | **Technique** | | **Metabolite** | | **LOD** | **Sensitivity** | **Ref** | |
|  | MoS_2_ | Electrochemical | | FET | | DNA hybridization | | 10 fM | 17 mV/dec | [184] | |
|  | Graphene | Electrolyte-Gated FET | | FET | | DNA hybridization | | 25 aM | 24 mV/dec | [192] | |
|  | Graphene | Liquid**-**gated FET | | FET | | DNA hybridization | | 1 pM | - | [232] | |
|  | Graphene | Liquid-gated FET | | FET | | DNA hybridization | | 10 nM to 500 nM | - | [183] | |
|  | Graphene | Multichannel FET | | FET | | DNA hybridization | | 10 pM | - | [233] | |
|  | Graphene | Multichannel FET | | FET | | DNA hybridization | | 100 fM | - | [234] | |
|  | Graphene | Gated FET | | FET | | DNA single-nucleotide polymorphism | | 10 pM to 1 nM | - | [235] | |
|  | MoS_2_ | (DNA)Bio-FET | | FET | | Doxorubicin (Anti-Cancer drug) | | 10^−4^ μM to 50 μM | 1.7 × 10^3^ A/A | [236] | |
|  | MoS_2_ | Electrolyte-gated FETs | | FET | | DNA fragments (chromosome 21 or 13) | | 1 fM | - | [237] | |
|  | Graphene | Electrolyte gated FET | | FET | | DNA hybridization | | 10 aM | 26.5 mV/dec | [238] | |
|  | Graphene | Multi-channel FET | | FET | | DNA hybridization | | 10 pM |  | [233] | |
|  | RGO | Liquid-gated FET | | FET | | peptide nucleic acid (PNA)–DNA hybridization | | 100 fM | - | [239] | |
|  | MoS_2_ | (DNA)Bio-FET | | FET | | prostate-specific antigen, PSA | | 1fg/ml | - | [139] | |
|  | MoS_2_ | (anti-PSA) Bio-FET | | FET | | prostate-specific antigen, PSA | | 1pg/ml | 4.3 V/dec | [240] | |
|  | MoS_2_ | (anti-PSA) Bio-FET | | FET | | prostate-specific antigen, PSA | | 3.75nM | - | [241] | |
|  | MoS_2_ | (anti-PSA) Bio-FET | | FET | | prostate-specific antigen, PSA | | 100 fg/mL | - | [242] | |
|  | Graphene | (anti-PSA) Bio-FET | | FET | | prostate-specific antigen, PSA | | 100 fg/mL | 20 mV/dec | [181] | |
|  | Graphene | (anti-PSA) Bio-FET | | FET | | prostate-specific antigen, PSA | | 1nM | - | [243] | |

**References**

[1] P.B. Luppa, C. Müller, A. Schlichtiger, H. Schlebusch, Point-of-care testing (POCT): Current techniques and future perspectives, TrAC Trends in Analytical Chemistry 30(6) (2011) 887-898.

[2] V. Renugopalakrishnan, T.N. Narayanan, S.D. Kumar, Y.C. Kudwa, J.R. Eswara, Two-dimensional nanomaterials for healthcare and lab-on-a-chip devices, MRS Communications 8(3) (2018) 625-626.

[3] W.K. Peng, D. Paesani, Omics meeting Onics: towards the next generation of spectroscopic-based technologies in personalized medicine, MDPI, 2019, p. 39.

[4] S. Konermann, P. Lotfy, N.J. Brideau, J. Oki, M.N. Shokhirev, P.D. Hsu, Transcriptome engineering with RNA-targeting type VI-D CRISPR effectors, Cell 173(3) (2018) 665-676. e14.

[5] F.D. Urnov, A path to efficient gene editing, Nature Medicine 24(7) (2018) 899-900.

[6] B. van Steensel, E.E. Furlong, The role of transcription in shaping the spatial organization of the genome, Nature reviews Molecular cell biology 20(6) (2019) 327-337.

[7] M.J. Rowley, V.G. Corces, Organizational principles of 3D genome architecture, Nature Reviews Genetics 19(12) (2018) 789-800.

[8] M. Chiasson, D.M. Fowler, Mutagenesis-based protein structure determination, Nature genetics 51(7) (2019) 1072-1073.

[9] W. Reik, Stability and flexibility of epigenetic gene regulation in mammalian development, Nature 447(7143) (2007) 425-432.

[10] R. Barouki, K. Audouze, X. Coumoul, F. Demenais, D. Gauguier, Integration of the human exposome with the human genome to advance medicine, Biochimie 152 (2018) 155-158.

[11] A. Dupré, K.-M. Lei, P.-I. Mak, R.P. Martins, W.K. Peng, Micro-and nanofabrication NMR technologies for point-of-care medical applications–a review, Microelectronic Engineering 209 (2019) 66-74.

[12] W.K. Peng, L. Chen, J. Han, Development of miniaturized, portable magnetic resonance relaxometry system for point-of-care medical diagnosis, Review of Scientific Instruments 83(9) (2012) 095115.

[13] R. Dias, A. Torkamani, Artificial intelligence in clinical and genomic diagnostics. Genome Med 11 (1): 70, 2019.

[14] S.K. Datta, C. Bonnet, A. Gyrard, R.P.F. Da Costa, K. Boudaoud, Applying Internet of Things for personalized healthcare in smart homes, 2015 24th Wireless and Optical Communication Conference (WOCC), IEEE, 2015, pp. 164-169.

[15] L. Zhu, M. Farhat, K.N. Salama, P.-Y. Chen, Two-dimensional materials-based radio frequency wireless communication and sensing systems for Internet-of-things applications, Emerging 2D Materials and Devices for the Internet of Things, Elsevier2020, pp. 29-57.

[16] M. Nolan, O. Sean, O, Callaghan, S.; Fagas, G.; Greer, JC; Frauenheim, T, Nano Lett 7(1) (2007) 34-38.

[17] A. St John, C.P. Price, Existing and emerging technologies for point-of-care testing, The Clinical Biochemist Reviews 35(3) (2014) 155.

[18] M. Xu, T. Liang, M. Shi, H. Chen, Graphene-like two-dimensional materials, Chemical reviews 113(5) (2013) 3766-3798.

[19] R. Rao, C.L. Pint, A.E. Islam, R.S. Weatherup, S. Hofmann, E.R. Meshot, F. Wu, C. Zhou, N. Dee, P.B. Amama, Carbon nanotubes and related nanomaterials: critical advances and challenges for synthesis toward mainstream commercial applications, ACS nano 12(12) (2018) 11756-11784.

[20] A. Bolotsky, D. Butler, C. Dong, K. Gerace, N.R. Glavin, C. Muratore, J.A. Robinson, A. Ebrahimi, Two-dimensional materials in biosensing and healthcare: from in vitro diagnostics to optogenetics and beyond, Acs Nano 13(9) (2019) 9781-9810.

[21] X. Ren, H. Ma, T. Zhang, Y. Zhang, T. Yan, B. Du, Q. Wei, Sulfur-doped graphene-based immunological biosensing platform for multianalysis of cancer biomarkers, ACS applied materials & interfaces 9(43) (2017) 37637-37644.

[22] J. Ji, J. Wen, Y. Shen, Y. Lv, Y. Chen, S. Liu, H. Ma, Y. Zhang, Simultaneous noncovalent modification and exfoliation of 2D carbon nitride for enhanced electrochemiluminescent biosensing, Journal of the American Chemical Society 139(34) (2017) 11698-11701.

[23] M. Li, C. Liu, H. Zhao, H. An, H. Cao, Y. Zhang, Z. Fan, Tuning sulfur doping in graphene for highly sensitive dopamine biosensors, Carbon 86 (2015) 197-206.

[24] V. Urbanová, F. Karlický, A. Matěj, F. Šembera, Z. Janoušek, J.A. Perman, V. Ranc, K. Čépe, J. Michl, M. Otyepka, Fluorinated graphenes as advanced biosensors–effect of fluorine coverage on electron transfer properties and adsorption of biomolecules, Nanoscale 8(24) (2016) 12134-12142.

[25] S. Zhang, R. Geryak, J. Geldmeier, S. Kim, V.V. Tsukruk, Synthesis, assembly, and applications of hybrid nanostructures for biosensing, Chemical reviews 117(20) (2017) 12942-13038.

[26] K. Shavanova, Y. Bakakina, I. Burkova, I. Shtepliuk, R. Viter, A. Ubelis, V. Beni, N. Starodub, R. Yakimova, V. Khranovskyy, Application of 2D non-graphene materials and 2D oxide nanostructures for biosensing technology, Sensors 16(2) (2016) 223.

[27] P.A. Harris, R. Taylor, B.L. Minor, V. Elliott, M. Fernandez, L. O'Neal, L. McLeod, G. Delacqua, F. Delacqua, J. Kirby, The REDCap consortium: Building an international community of software platform partners, Journal of biomedical informatics 95 (2019) 103208.

[28] E. Primiceri, M.S. Chiriacò, F.M. Notarangelo, A. Crocamo, D. Ardissino, M. Cereda, A.P. Bramanti, M.A. Bianchessi, G. Giannelli, G. Maruccio, Key enabling technologies for point-of-care diagnostics, Sensors 18(11) (2018) 3607.

[29] F.R. Vogenberg, C.I. Barash, M. Pursel, Personalized medicine: part 1: evolution and development into theranostics, Pharmacy and Therapeutics 35(10) (2010) 560.

[30] O. Stegle, F.P. Roth, Q. Morris, J. Listgarten, Personalized Medicine: From genotypes and molecular phenotypes towards computed therapy, Biocomputing 2012, World Scientific2012, pp. 323-326.

[31] W.K. Peng, D. Paesani, Omics Meeting Onics: Towards the Next Generation of Spectroscopic-Based Technologies in Personalized Medicine, Journal of Personalized Medicine 9(3) (2019) 39.

[32] A. Cruz, W.K. Peng, Perspective: Cellular and Molecular Profiling Technologies in Personalized Oncology, Journal of Personalized Medicine 9(3) (2019) 44.

[33] R.H. Horton, A.M. Lucassen, Recent developments in genetic/genomic medicine, Clinical Science 133(5) (2019) 697-708.

[34] S. Purcell, B. Neale, K. Todd-Brown, L. Thomas, M.A. Ferreira, D. Bender, J. Maller, P. Sklar, P.I. De Bakker, M.J. Daly, PLINK: a tool set for whole-genome association and population-based linkage analyses, The American journal of human genetics 81(3) (2007) 559-575.

[35] K. Karczewski, L. Francioli, G. Tiao, B. Cummings, J. Alföldi, Q. Wang, Genome Aggregation Database, C.(2020), The mutational constraint spectrum quantified from variation in 141 434-443.

[36] E.D. Green, C. Gunter, L.G. Biesecker, V. Di Francesco, C.L. Easter, E.A. Feingold, A.L. Felsenfeld, D.J. Kaufman, E.A. Ostrander, W.J. Pavan, Strategic vision for improving human health at The Forefront of Genomics, Nature 586(7831) (2020) 683-692.

[37] K. Strimbu, J. Tavel, The maturometer-Instrumental test and redesign, Curr Opin HIV AIDS 5(6) (2010) 463-6.

[38] E.R. Mardis, The impact of next-generation sequencing on cancer genomics: from discovery to clinic, Cold Spring Harbor Perspectives in Medicine 9(9) (2019) a036269.

[39] M. Vrijheid, The exposome: a new paradigm to study the impact of environment on health, Thorax 69(9) (2014) 876-878.

[40] P. Vineis, O. Robinson, M. Chadeau-Hyam, A. Dehghan, I. Mudway, S. Dagnino, What is new in the exposome?, Environment international 143 (2020) 105887.

[41] W.K. Peng, T.F. Kong, C.S. Ng, L. Chen, Y. Huang, A.A.S. Bhagat, N.-T. Nguyen, P.R. Preiser, J. Han, Micromagnetic resonance relaxometry for rapid label-free malaria diagnosis, Nature medicine 20(9) (2014) 1069-1073.

[42] M.I. Veiga, W.K. Peng, Rapid phenotyping towards personalized malaria medicine, Malaria Journal 19(1) (2020) 1-5.

[43] W.K. Peng, Clustering Nuclear Magnetic Resonance: Machine learning assistive rapid two‐dimensional relaxometry mapping, Engineering Reports 3(10) (2021) e12383.

[44] J.L. Pépin, S. Bailly, R. Tamisier, Incorporating polysomnography into obstructive sleep apnoea phenotyping: moving towards personalised medicine for OSA, BMJ Publishing Group Ltd, 2018, pp. 409-411.

[45] J.-P. Onnela, Opportunities and challenges in the collection and analysis of digital phenotyping data, Neuropsychopharmacology 46(1) (2021) 45-54.

[46] J. Torous, M.V. Kiang, J. Lorme, J.-P. Onnela, New tools for new research in psychiatry: a scalable and customizable platform to empower data driven smartphone research, JMIR mental health 3(2) (2016) e5165.

[47] J. Blom, C. Benatti, C. Colliva, F. Tascedda, L. Pani, Digital phenotyping and dynamic monitoring of adolescents treated for cancer to guide intervention: embracing a new era, Frontiers in oncology 11 (2021) 2397.

[48] J.S. Talboom, M.J. Huentelman, Big data collision: the internet of things, wearable devices and genomics in the study of neurological traits and disease, Human molecular genetics 27(R1) (2018) R35-R39.

[49] P.A. Kavsak, N. Zielinski, D. Li, P.J. McNamara, K. Adeli, Challenges of implementing Point-of-Care Testing (POCT) glucose meters in a pediatric acute care setting, Clinical Biochemistry 37(9) (2004) 811-817.

[50] S.J. Aronson, H.L. Rehm, Building the foundation for genomics in precision medicine, Nature 526(7573) (2015) 336-42.

[51] H.L. Rehm, Evolving health care through personal genomics, Nature Reviews Genetics 18(4) (2017) 259-267.

[52] J.M. Heather, B. Chain, The sequence of sequencers: The history of sequencing DNA, Genomics 107(1) (2016) 1-8.

[53] M. Snyder, Genomics and personalized medicine: what everyone needs to know, Oxford University Press2016.

[54] C.-Z. Zhang, A. Spektor, H. Cornils, J.M. Francis, E.K. Jackson, S. Liu, M. Meyerson, D. Pellman, Chromothripsis from DNA damage in micronuclei, Nature 522(7555) (2015) 179-184.

[55] S. Nik-Zainal, P. Van Loo, D. Wedge, L. Alexandrov, C. Greenman, K. Lau, K. Raine, D. Jones, J. Marshall, M. Ramakrishna, Breast Cancer Working Group of the International Cancer Genome, C2012, The life history of 21 994-1007.

[56] P. Suwinski, C. Ong, M.H. Ling, Y.M. Poh, A.M. Khan, H.S. Ong, Advancing personalized medicine through the application of whole exome sequencing and big data analytics, Frontiers in genetics 10 (2019) 49.

[57] X. Zhou, S.H. Lee, An integrative analysis of genomic and exposomic data for complex traits and phenotypic prediction, Scientific reports 11(1) (2021) 1-16.

[58] X. Hu, D.I. Walker, Y. Liang, M.R. Smith, M.L. Orr, B.D. Juran, C. Ma, K. Uppal, M. Koval, G.S. Martin, A scalable workflow to characterize the human exposome, Nature communications 12(1) (2021) 1-12.

[59] J. Adler-Milstein, A.K. Jha, HITECH Act drove large gains in hospital electronic health record adoption, Health affairs 36(8) (2017) 1416-1422.

[60] P. Georgiou, C. Toumazou, Semiconductors for early detection and therapy, Electronics letters 47(26) (2011) 4-6.

[61] T. Vo-Dinh, Biomedical photonics handbook: biomedical diagnostics, CRC press2014.

[62] Y. Miyamoto, Genome technology and electronics, OKI Technical Review 70 (2003) 82-85.

[63] R. Sommer, C. Nelson, A. Greenquist, Dry-reagent strips for measuring phenytoin in serum, Clinical chemistry 32(9) (1986) 1770-1774.

[64] J.B. Haun, C.M. Castro, R. Wang, V.M. Peterson, B.S. Marinelli, H. Lee, R. Weissleder, Micro-NMR for rapid molecular analysis of human tumor samples, Science translational medicine 3(71) (2011) 71ra16-71ra16.

[65] W.K. Peng, T.-T. Ng, T.P. Loh, Machine learning assistive rapid, label-free molecular phenotyping of blood with two-dimensional NMR correlational spectroscopy, Communications biology 3(1) (2020) 1-10.

[66] W.K. Peng, L. Chen, B.O. Boehm, J. Han, T.P. Loh, Molecular phenotyping of oxidative stress in diabetes mellitus with point-of-care NMR system, NPJ aging and mechanisms of disease 6(1) (2020) 1-12.

[67] X. Wang, W. Peng, W. Lew, Flux-closure chirality control and domain wall trapping in asymmetric magnetic ring, Journal of Applied Physics 106(4) (2009) 043905.

[68] M.U. Gami, D. Raji Pillai, S. Cherian, Emerging Technologies for Point-of-Care Testing: A future outlook for Scientists and Engineers.

[69] C.M. Pandey, S. Augustine, S. Kumar, S. Kumar, S. Nara, S. Srivastava, B.D. Malhotra, Microfluidics based point‐of‐care diagnostics, Biotechnology journal 13(1) (2018) 1700047.

[70] A. Vinaiphat, J.K. Low, K.W. Yeoh, W.J. Chng, S.K. Sze, Application of advanced mass spectrometry-based proteomics to study hypoxia driven cancer progression, Frontiers in Oncology 11 (2021) 98.

[71] D.N. Perkins, D.J. Pappin, D.M. Creasy, J.S. Cottrell, Probability‐based protein identification by searching sequence databases using mass spectrometry data, ELECTROPHORESIS: An International Journal 20(18) (1999) 3551-3567.

[72] J. Samoilova, M. Matveeva, O. Tonkih, D. Kudlau, O. Oleynik, A. Kanev, A prospective study: Highlights of hippocampal spectroscopy in cognitive impairment in patients with type 1 and type 2 diabetes, Journal of Personalized Medicine 11(2) (2021) 148.

[73] L. Li, J. Wu, L. Yang, H. Wang, Y. Xu, K. Shen, Fourier Transform Infrared Spectroscopy: An Innovative Method for the Diagnosis of Ovarian Cancer, Cancer Management and Research 13 (2021) 2389.

[74] W. Gao, S. Emaminejad, H.Y.Y. Nyein, S. Challa, K. Chen, A. Peck, H.M. Fahad, H. Ota, H. Shiraki, D. Kiriya, Fully integrated wearable sensor arrays for multiplexed in situ perspiration analysis, Nature 529(7587) (2016) 509-514.

[75] M. Bariya, H.Y.Y. Nyein, A. Javey, Wearable sweat sensors, Nature Electronics 1(3) (2018) 160-171.

[76] R.M. Torrente-Rodríguez, J. Tu, Y. Yang, J. Min, M. Wang, Y. Song, Y. Yu, C. Xu, C. Ye, W.W. IsHak, Investigation of cortisol dynamics in human sweat using a graphene-based wireless mHealth system, Matter 2(4) (2020) 921-937.

[77] M.K. Sung, S. Singh, M.K. Kalra, Current status of low dose multi-detector CT in the urinary tract, World Journal of Radiology 3(11) (2011) 256.

[78] L. Martin, R. Ruddlesden, C. Makepeace, L. Robinson, T. Mistry, H. Starritt, Paediatric x-ray radiation dose reduction and image quality analysis, Journal of Radiological Protection 33(3) (2013) 621.

[79] Y. Huang, A.A. Liu, B. Lafon, D. Friedman, M. Dayan, X. Wang, M. Bikson, W.K. Doyle, O. Devinsky, L.C. Parra, Measurements and models of electric fields in the in vivo human brain during transcranial electric stimulation, elife 6 (2017) e18834.

[80] N. Noury, J.F. Hipp, M. Siegel, Physiological processes non-linearly affect electrophysiological recordings during transcranial electric stimulation, Neuroimage 140 (2016) 99-109.

[81] W.T. Sow, F. Ye, C. Zhang, H. Li, Smart materials for point-of-care testing: From sample extraction to analyte sensing and readout signal generator, Biosensors and Bioelectronics 170 (2020) 112682.

[82] E. Petryayeva, W.R. Algar, Toward point-of-care diagnostics with consumer electronic devices: the expanding role of nanoparticles, Rsc Advances 5(28) (2015) 22256-22282.

[83] L. Huerta-Nuñez, G. Gutierrez-Iglesias, A. Martinez-Cuazitl, M. Mata-Miranda, V. Alvarez-Jiménez, V. Sánchez-Monroy, A. Golberg, C. González-Díaz, A biosensor capable of identifying low quantities of breast cancer cells by electrical impedance spectroscopy, Scientific reports 9(1) (2019) 1-12.

[84] H.J. Parab, C. Jung, J.-H. Lee, H.G. Park, A gold nanorod-based optical DNA biosensor for the diagnosis of pathogens, Biosensors and Bioelectronics 26(2) (2010) 667-673.

[85] J. Kim, A. Campbell, De vila, BE-F., and Wang, J.(2019). Wearable biosensors for healthcare monitoring, Nat. Biotechnol 37 389-406.

[86] A. Ozcan, U. Demirci, Ultra wide-field lens-free monitoring of cells on-chip, Lab on a Chip 8(1) (2008) 98-106.

[87] D.S. Boyle, K.R. Hawkins, M.S. Steele, M. Singhal, X. Cheng, Emerging technologies for point-of-care CD4 T-lymphocyte counting, Trends in biotechnology 30(1) (2012) 45-54.

[88] B.D. DeBusschere, G.T. Kovacs, Portable cell-based biosensor system using integrated CMOS cell-cartridges, Biosensors and Bioelectronics 16(7-8) (2001) 543-556.

[89] A. Tretyakov, A. Steube, H.P. Saluz, T. Schenk, High-intensity UV laser ChIP-seq for the study of protein-DNA interactions in living cells, Nature Communications 8(1) (2017).

[90] N.A. Pchelintsev, P.D. Adams, D.M. Nelson, Critical parameters for efficient sonication and improved chromatin immunoprecipitation of high molecular weight proteins, PloS one 11(1) (2016) e0148023.

[91] A. Nebbioso, R. Benedetti, M. Conte, V. Carafa, F. De Bellis, J. Shaik, F. Matarese, B. Della Ventura, F. Gesuele, R. Velotta, Time-resolved analysis of DNA-protein interactions in living cells by UV laser pulses, Scientific reports 7(1) (2017) 1-13.

[92] P.D. Schoppee Bortz, B.R. Wamhoff, Chromatin immunoprecipitation (ChIP): revisiting the efficacy of sample preparation, sonication, quantification of sheared DNA, and analysis via PCR, PloS one 6(10) (2011) e26015.

[93] Y.J. Yuan, M.J. Van der Werff, W. Xu, A field-programmable-gate-array-based high-speed transceiver for a quartz crystal microbalance induced bond-rupture sensor, Measurement Science and Technology 22(4) (2011) 045201.

[94] M.K. Park, Q. Liu, K.W. Kim, Y. Shin, J.S. Kee, J. Song, G.-Q. Lo, D.-L. Kwong, Integrated silicon microring resonator devices for point-of-care diagnostic applications, Silicon Photonics IX, SPIE, 2014, pp. 182-189.

[95] T.F. Kong, W.K. Peng, T.D. Luong, N.-T. Nguyen, J. Han, Adhesive-based liquid metal radio-frequency microcoil for magnetic resonance relaxometry measurement, Lab on a Chip 12(2) (2012) 287-294.

[96] G. Guan, P.C. Chen, W.K. Peng, A.A. Bhagat, C.J. Ong, J. Han, Real-time control of a microfluidic channel for size-independent deformability cytometry, Journal of Micromechanics and Microengineering 22(10) (2012) 105037.

[97] S. Saska, L. Pilatti, A. Blay, J.A. Shibli, Bioresorbable polymers: Advanced materials and 4D printing for tissue engineering, Polymers 13(4) (2021) 563.

[98] S.H. Ang, M. Rambeli, T.M. Thevarajah, Y.B. Alias, S.M. Khor, Quantitative, single-step dual measurement of hemoglobin A1c and total hemoglobin in human whole blood using a gold sandwich immunochromatographic assay for personalized medicine, Biosensors and Bioelectronics 78 (2016) 187-193.

[99] C. Liu, Q. Jia, C. Yang, R. Qiao, L. Jing, L. Wang, C. Xu, M. Gao, Lateral flow immunochromatographic assay for sensitive pesticide detection by using Fe3O4 nanoparticle aggregates as color reagents, Analytical chemistry 83(17) (2011) 6778-6784.

[100] L. Anfossi, F. Di Nardo, C. Giovannoli, C. Passini, C. Baggiani, Increased sensitivity of lateral flow immunoassay for ochratoxin A through silver enhancement, Analytical and bioanalytical chemistry 405(30) (2013) 9859-9867.

[101] G. Ertürk, B. Mattiasson, Molecular imprinting techniques used for the preparation of biosensors, Sensors 17(2) (2017) 288.

[102] G. Selvolini, G. Marrazza, MIP-based sensors: promising new tools for cancer biomarker determination, Sensors 17(4) (2017) 718.

[103] A.H. Safaryan, A.M. Smith, T.S. Bedwell, E.V. Piletska, F. Canfarotta, S.A. Piletsky, Optimisation of the preservation conditions for molecularly imprinted polymer nanoparticles specific for trypsin, Nanoscale Advances 1(9) (2019) 3709-3714.

[104] H.A. Dewia, B.S. Fangben Mengb, C. Guoa, B. Norlingc, X. Chenb, S. Lima, RSC Advances RSCPublishing.

[105] D.J. Denmark, R.H. Hyde, C. Gladney, M.-H. Phan, K.S. Bisht, H. Srikanth, P. Mukherjee, S. Witanachchi, Photopolymerization-based synthesis of iron oxide nanoparticle embedded PNIPAM nanogels for biomedical applications, Drug Delivery 24(1) (2017) 1317-1324.

[106] B.-K. Lim, E.C. Tighe, S.D. Kong, The use of magnetic targeting for drug delivery into cardiac myocytes, Journal of Magnetism and Magnetic Materials 473 (2019) 21-25.

[107] K.-B. Kim, Y.W. Kim, S.K. Lim, T.H. Roh, D.Y. Bang, S.M. Choi, D.S. Lim, Y.J. Kim, S.-H. Baek, M.-K. Kim, Risk assessment of zinc oxide, a cosmetic ingredient used as a UV filter of sunscreens, Journal of Toxicology and Environmental Health, Part B 20(3) (2017) 155-182.

[108] P. Sanches, W. Souza, S. Gemini-Piperni, A. Rossi, S. Scapin, V. Midlej, Y. Sade, A.P. Leme, M. Benchimol, L. Rocha, Rutile nano–bio-interactions mediate dissimilar intracellular destiny in human skin cells, Nanoscale Advances 1(6) (2019) 2216-2228.

[109] X. He, J.-T. Hou, X. Sun, P. Jangili, J. An, Y. Qian, J.S. Kim, J. Shen, NIR-II Photo-Amplified Sonodynamic Therapy Using Sodium Molybdenum Bronze Nanoplatform against Subcutaneous Staphylococcus Aureus Infection, Advanced Functional Materials n/a(n/a) 2203964.

[110] M. Mohammadniaei, H.V. Nguyen, M.V. Tieu, M.-H. Lee, 2D materials in development of electrochemical point-of-care cancer screening devices, Micromachines 10(10) (2019) 662.

[111] J. Zheng, J. Li, L. Zhang, X. Chen, Y. Yu, H. Huang, Post-graphene 2D materials-based antimicrobial agents: focus on fabrication strategies and biosafety assessments, Journal of Materials Science 55(17) (2020) 7226-7246.

[112] M. Devi, Application of 2D Nanomaterials as Fluorescent Biosensors, Adapting 2D Nanomaterials for Advanced Applications, ACS Publications2020, pp. 117-141.

[113] D. Akinwande, C.J. Brennan, J.S. Bunch, P. Egberts, J.R. Felts, H. Gao, R. Huang, J.-S. Kim, T. Li, Y. Li, A review on mechanics and mechanical properties of 2D materials—Graphene and beyond, Extreme Mechanics Letters 13 (2017) 42-77.

[114] Q. Ma, G. Ren, K. Xu, J.Z. Ou, Tunable optical properties of 2D materials and their applications, Advanced Optical Materials 9(2) (2021) 2001313.

[115] P. Bøggild, D.M. Mackenzie, P.R. Whelan, D.H. Petersen, J.D. Buron, A. Zurutuza, J. Gallop, L. Hao, P.U. Jepsen, Mapping the electrical properties of large-area graphene, 2D Materials 4(4) (2017) 042003.

[116] C. Anichini, W. Czepa, D. Pakulski, A. Aliprandi, A. Ciesielski, P. Samorì, Chemical sensing with 2D materials, Chemical Society Reviews 47(13) (2018) 4860-4908.

[117] H. Zhang, Introduction: 2D materials chemistry, ACS Publications, 2018, pp. 6089-6090.

[118] R.J. Toh, W.K. Peng, J. Han, M. Pumera, Haemoglobin electrochemical detection on various reduced graphene surfaces: well-defined glassy carbon electrode outperforms the graphenoids, RSC Advances 4(16) (2014) 8050-8054.

[119] R.J. Toh, W.K. Peng, J. Han, M. Pumera, Direct in vivo electrochemical detection of haemoglobin in red blood cells, Scientific reports 4(1) (2014) 1-6.

[120] C. Choi, Y. Lee, K.W. Cho, J.H. Koo, D.-H. Kim, Wearable and implantable soft bioelectronics using two-dimensional materials, Accounts of chemical research 52(1) (2018) 73-81.

[121] Y.-M. Lin, C. Dimitrakopoulos, K.A. Jenkins, D.B. Farmer, H.-Y. Chiu, A. Grill, P. Avouris, 100-GHz transistors from wafer-scale epitaxial graphene, Science 327(5966) (2010) 662-662.

[122] F. Reale, K. Sharda, C. Mattevi, From bulk crystals to atomically thin layers of group VI-transition metal dichalcogenides vapour phase synthesis, Applied Materials Today 3 (2016) 11-22.

[123] N. Rohaizad, C.C. Mayorga-Martinez, M. Fojtů, N.M. Latiff, M. Pumera, Two-dimensional materials in biomedical, biosensing and sensing applications, Chemical Society Reviews 50(1) (2021) 619-657.

[124] G. Ramalingam, P. Kathirgamanathan, G. Ravi, T. Elangovan, kumar BA, Manivannan N., Kasinathan K, Quantum confinement effect of 2D nanomaterials. Quantum Dots Fundam. Appl (2020).

[125] D. Du, Z. Zou, Y. Shin, J. Wang, H. Wu, M.H. Engelhard, J. Liu, I.A. Aksay, Y. Lin, Sensitive immunosensor for cancer biomarker based on dual signal amplification strategy of graphene sheets and multienzyme functionalized carbon nanospheres, Analytical chemistry 82(7) (2010) 2989-2995.

[126] Z. Ao, J. Yang, S. Li, Q. Jiang, Enhancement of CO detection in Al doped graphene, Chemical Physics Letters 461(4-6) (2008) 276-279.

[127] D. Stankovich, Dikin, GHB Dommett, KM Kohlhaas, EJ Zimney, EA Stach, RD Piner, ST Nguyen, and RS Ruoff, Nature 442 (2006) 282.

[128] A.E. Moutaouakil, M. Belmoubarik, W.K. Peng, Graphene in the fight against malaria, arXiv preprint arXiv:2008.13605 (2020).

[129] A.E. Moutaouakil, S. Poovathy, M. Belmoubarik, W.K. Peng, Graphene-based biosensor for Viral Detection, arXiv preprint arXiv:2006.11881 (2020).

[130] L. Liang, J. Wang, W. Lin, B.G. Sumpter, V. Meunier, M. Pan, Electronic bandgap and edge reconstruction in phosphorene materials, Nano letters 14(11) (2014) 6400-6406.

[131] M.I. Katsnelson, Graphene: Carbon in Two Dimensions, Cambridge University Press, Cambridge, 2012.

[132] X. Kang, J. Wang, H. Wu, I.A. Aksay, J. Liu, Y. Lin, Glucose oxidase–graphene–chitosan modified electrode for direct electrochemistry and glucose sensing, Biosensors and Bioelectronics 25(4) (2009) 901-905.

[133] N. Peres, F. Klironomos, S.-W. Tsai, J. Santos, J.L. Dos Santos, A.C. Neto, Electron waves in chemically substituted graphene, EPL (Europhysics Letters) 80(6) (2007) 67007.

[134] H. Chen, X. He, Z. Zhou, Z. Wu, H. Li, X. Peng, Y. Zhou, C. Tan, J. Shen, Metallic phase enabling MoS2 nanosheets as an efficient sonosensitizer for photothermal-enhanced sonodynamic antibacterial therapy, Journal of Nanobiotechnology 20(1) (2022) 136.

[135] X. Li, H. Zhu, Two-dimensional MoS2: Properties, preparation, and applications, Journal of Materiomics 1(1) (2015) 33-44.

[136] P. Yadav, Z. Cao, A. Barati Farimani, DNA Detection with Single-Layer Ti3C2 MXene Nanopore, ACS nano 15(3) (2021) 4861-4869.

[137] J. Prasongkit, Ultra-fast DNA sequencing based on Mxenes.

[138] V. Shukla, N.K. Jena, A. Grigoriev, R. Ahuja, Prospects of graphene–hBN heterostructure nanogap for DNA sequencing, ACS applied materials & interfaces 9(46) (2017) 39945-39952.

[139] Y. Zhang, D. Feng, Y. Xu, Z. Yin, W. Dou, U.E. Habiba, C. Pan, Z. Zhang, H. Mou, H. Deng, DNA-based functionalization of two-dimensional MoS2 FET biosensor for ultrasensitive detection of PSA, Applied Surface Science 548 (2021) 149169.

[140] Y. Lv, X. Qin, K. Hu, Y. Huang, S. Zhao, Hybrid MoS2/g-C3N4-assisted LDI mass spectrometry for rapid detection of small molecules and polyethylene glycols and direct determination of uric acid in complicated biological samples, Microchimica Acta 188(1) (2021) 1-11.

[141] X. Zhou, S.H. Lee, An integrative analysis of genomic and exposomic data for complex traits and phenotypic prediction, Sci Rep 11(1) (2021) 21495.

[142] M. Berner, U. Hilbig, M.B. Schubert, G. Gauglitz, Laser-induced fluorescence detection platform for point-of-care testing, Measurement Science and Technology 28(8) (2017) 085701.

[143] R. Summers, S. Hu, Innovations towards personalised biomedical photonic devices, Measurement and Control 44(6) (2011) 186-189.

[144] J. Liu, H. Wang, X. Li, H. Chen, Z. Zhang, W. Pan, G. Luo, C. Yuan, Y. Ren, W. Lei, Ultrasensitive flexible near-infrared photodetectors based on Van der Waals Bi2Te3 nanoplates, Applied Surface Science 484 (2019) 542-550.

[145] K. Zhang, L. Zhang, L. Han, L. Wang, Z. Chen, H. Xing, X. Chen, Recent progress and challenges based on two-dimensional material photodetectors, Nano Express 2(1) (2021) 012001.

[146] J. Wang, W. Hu, Recent progress on integrating two-dimensional materials with ferroelectrics for memory devices and photodetectors, Chinese Physics B 26(3) (2017) 037106.

[147] L. Britnell, R. Ribeiro, a. Eckmann, R. Jalil, B. D, Belle, a. Mishchenko, Y.-J. Kim, RV Gorbachev, T. Georgiou, S. V. Morozov, a. N. Grigorenko, a. K. Geim, C. Casiraghi, a. H. Castro Neto, and K. S. Novoselov," Strong light-matter interactions in heterostructures of atomically thin films.," Science (New York, N. Y.) 340 (2013) 1311-4.

[148] H. Zhang, K.-K. Liu, Optical tweezers for single cells, Journal of the Royal Society interface 5(24) (2008) 671-690.

[149] C. Monat, P. Domachuk, C. Grillet, M. Collins, B. Eggleton, M. Cronin-Golomb, S. Mutzenich, T. Mahmud, G. Rosengarten, A. Mitchell, Optofluidics: a novel generation of reconfigurable and adaptive compact architectures, Microfluidics and Nanofluidics 4(1) (2008) 81-95.

[150] P. Pinapati, J.P. Joby, S. Cherukulappurath, Graphene Oxide Based Two-Dimensional Optical Tweezers for Low Power Trapping of Quantum Dots and E. coli Bacteria, ACS Applied Nano Materials 3(6) (2020) 5107-5115.

[151] H.H. Nguyen, J. Park, S. Kang, M. Kim, Surface Plasmon Resonance: A Versatile Technique for Biosensor Applications, Sensors 15(5) (2015) 10481-10510.

[152] H. Zhang, X. Li, F. He, M. Zhao, L. Ling, Turn-off colorimetric sensor for sequence-specific recognition of single-stranded DNA based upon Y-shaped DNA structure, Scientific Reports 8(1) (2018) 1-8.

[153] S. Catalán-Gómez, M. Briones, S. Cortijo-Campos, T. García-Mendiola, A. de Andrés, S. Garg, P. Kung, E. Lorenzo, J.L. Pau, A. Redondo-Cubero, Breast cancer biomarker detection through the photoluminescence of epitaxial monolayer MoS2 flakes, Scientific Reports 10(1) (2020) 1-9.

[154] A.A. Yanik, M. Huang, O. Kamohara, A. Artar, T.W. Geisbert, J.H. Connor, H. Altug, An optofluidic nanoplasmonic biosensor for direct detection of live viruses from biological media, Nano letters 10(12) (2010) 4962-4969.

[155] Y. Yang, Y. Song, X. Bo, J. Min, O.S. Pak, L. Zhu, M. Wang, J. Tu, A. Kogan, H. Zhang, A laser-engraved wearable sensor for sensitive detection of uric acid and tyrosine in sweat, Nature biotechnology 38(2) (2020) 217-224.

[156] S. Kaushik, U.K. Tiwari, A. Deep, R.K. Sinha, Two-dimensional transition metal dichalcogenides assisted biofunctionalized optical fiber SPR biosensor for efficient and rapid detection of bovine serum albumin, Scientific reports 9(1) (2019) 1-11.

[157] M. Lobry, D. Lahem, M. Loyez, M. Debliquy, K. Chah, M. David, C. Caucheteur, Non-enzymatic D-glucose plasmonic optical fiber grating biosensor, Biosensors and Bioelectronics 142 (2019) 111506.

[158] T. Patriarchi, A. Mohebi, J. Sun, A. Marley, R. Liang, C. Dong, K. Puhger, G.O. Mizuno, C.M. Davis, B. Wiltgen, An expanded palette of dopamine sensors for multiplex imaging in vivo, Nature methods 17(11) (2020) 1147-1155.

[159] D. Chen, H. Feng, J. Li, Graphene oxide: preparation, functionalization, and electrochemical applications, Chemical reviews 112(11) (2012) 6027-6053.

[160] W. Nie, Q. Wang, X. Yang, H. Zhang, Z. Li, L. Gao, Y. Zheng, X. Liu, K. Wang, High sensitivity surface plasmon resonance biosensor for detection of microRNA based on gold nanoparticles-decorated molybdenum sulfide, Analytica Chimica Acta 993 (2017) 55-62.

[161] L. Wu, C. Huang, B.P. Emery, A.C. Sedgwick, S.D. Bull, X.-P. He, H. Tian, J. Yoon, J.L. Sessler, T.D. James, Förster resonance energy transfer (FRET)-based small-molecule sensors and imaging agents, Chemical Society Reviews 49(15) (2020) 5110-5139.

[162] M. Tebyetekerwa, J. Zhang, Z. Xu, T.N. Truong, Z. Yin, Y. Lu, S. Ramakrishna, D. Macdonald, H.T. Nguyen, Mechanisms and applications of steady-state photoluminescence spectroscopy in two-dimensional transition-metal dichalcogenides, ACS nano 14(11) (2020) 14579-14604.

[163] B. Dong, H. Li, J. Sun, G.M. Mari, J. Ai, D. Han, X. Yu, K. Wen, J. Shen, Z. Wang, Homogeneous fluorescent immunoassay for the simultaneous detection of chloramphenicol and amantadine via the duplex FRET between carbon dots and WS2 nanosheets, Food Chemistry 327 (2020) 127107.

[164] C. Zhu, Z. Zeng, H. Li, F. Li, C. Fan, H. Zhang, Single-layer MoS2-based nanoprobes for homogeneous detection of biomolecules, Journal of the American Chemical Society 135(16) (2013) 5998-6001.

[165] Y. Zhang, B. Zheng, C. Zhu, X. Zhang, C. Tan, H. Li, B. Chen, J. Yang, J. Chen, Y. Huang, Single‐layer transition metal dichalcogenide nanosheet‐based nanosensors for rapid, sensitive, and multiplexed detection of DNA, Advanced Materials 27(5) (2015) 935-939.

[166] X. Liu, Y. Hou, S. Chen, J. Liu, Controlling dopamine binding by the new aptamer for a FRET-based biosensor, Biosensors and Bioelectronics 173 (2021) 112798.

[167] A. Raja, A.s. Montoya− Castillo, J. Zultak, X.-X. Zhang, Z. Ye, C. Roquelet, D.A. Chenet, A.M. Van Der Zande, P. Huang, S. Jockusch, Energy transfer from quantum dots to graphene and MoS2: The role of absorption and screening in two-dimensional materials, Nano letters 16(4) (2016) 2328-2333.

[168] V. Baptista, W.K. Peng, G. Minas, M.I. Veiga, S.O. Catarino, Review of Microdevices for Hemozoin-Based Malaria Detection, Biosensors 12(2) (2022) 110.

[169] B.J. Walder, C. Berk, W.-C. Liao, A.J. Rossini, M. Schwarzwälder, U. Pradere, J. Hall, A. Lesage, C. Copéret, L. Emsley, One-and two-dimensional high-resolution NMR from flat surfaces, ACS central science 5(3) (2019) 515-523.

[170] W.K. Peng, K. Takeda, Efficient cross polarization with simultaneous adiabatic frequency sweep on the source and target channels, Journal of Magnetic Resonance 188(2) (2007) 267-274.

[171] V. Egelhofer, J. Gobom, H. Seitz, P. Giavalisco, H. Lehrach, E. Nordhoff, Protein identification by MALDI-TOF-MS peptide mapping: a new strategy, Analytical Chemistry 74(8) (2002) 1760-1771.

[172] P. Chaurand, F. Luetzenkirchen, B. Spengler, Peptide and protein identification by matrix-assisted laser desorption ionization (MALDI) and MALDI-post-source decay time-of-flight mass spectrometry, Journal of the American Society for Mass Spectrometry 10(2) (1999) 91-103.

[173] T.A. Shaler, C.H. Becker, Y. Tan, J.N. Wickham, K.J. Wu, Analysis of enzymatic DNA sequencing reactions by matrix‐assisted laser desorption/ionization time‐of‐flight mass spectrometry, Rapid communications in mass spectrometry 9(10) (1995) 942-947.

[174] N.R. Glavin, A. Bolotsky, D.J. Butler, C. Dong, K. Gerace, J.A. Robinson, A. Ebrahimi, C. Muratore, Two-Dimensional Materials in Biosensing and Healthcare: From In Vitro Diagnostics to Optogenetics and Beyond (Postprint), (2019).

[175] J.H. An, S.J. Park, O.S. Kwon, J. Bae, J. Jang, High-performance flexible graphene aptasensor for mercury detection in mussels, ACS nano 7(12) (2013) 10563-10571.

[176] P. Li, B. Liu, D. Zhang, Y.e. Sun, J. Liu, Graphene field-effect transistors with tunable sensitivity for high performance Hg (II) sensing, Applied Physics Letters 109(15) (2016) 153101.

[177] T. Scientific, Thomas Brand Mercury Ion Electrodes, 2019.

[178] P. Li, D. Zhang, C. Jiang, X. Zong, Y. Cao, Ultra-sensitive suspended atomically thin-layered black phosphorus mercury sensors, Biosensors and Bioelectronics 98 (2017) 68-75.

[179] N. Hasan, B. Hou, A.L. Moore, A.D. Radadia, Enhanced ionic sensitivity in solution‐gated graphene‐hexagonal boron nitride heterostructure field‐effect transistors, Advanced Materials Technologies 3(8) (2018) 1800133.

[180] Z. Cheng, J. Hou, Q. Zhou, T. Li, H. Li, L. Yang, K. Jiang, C. Wang, Y. Li, Y. Fang, Sensitivity limits and scaling of bioelectronic graphene transducers, Nano letters 13(6) (2013) 2902-2907.

[181] D.-J. Kim, I.Y. Sohn, J.-H. Jung, O.J. Yoon, N.-E. Lee, J.-S. Park, Reduced graphene oxide field-effect transistor for label-free femtomolar protein detection, Biosensors and bioelectronics 41 (2013) 621-626.

[182] Z. Cheng, Q. Li, Z. Li, Q. Zhou, Y. Fang, Suspended graphene sensors with improved signal and reduced noise, Nano letters 10(5) (2010) 1864-1868.

[183] X. Dong, Y. Shi, W. Huang, P. Chen, L.J. Li, Electrical detection of DNA hybridization with single‐base specificity using transistors based on CVD‐grown graphene sheets, Advanced Materials 22(14) (2010) 1649-1653.

[184] D.-W. Lee, J. Lee, I.Y. Sohn, B.-Y. Kim, Y.M. Son, H. Bark, J. Jung, M. Choi, T.H. Kim, C. Lee, Field-effect transistor with a chemically synthesized MoS2 sensing channel for label-free and highly sensitive electrical detection of DNA hybridization, Nano Research 8(7) (2015) 2340-2350.

[185] J.D. Newman, A.P. Turner, Home blood glucose biosensors: a commercial perspective, Biosensors and bioelectronics 20(12) (2005) 2435-2453.

[186] T. Tu, B. Liang, Q. Cao, L. Fang, Q. Zhu, Y. Cai, X. Ye, Fully transient electrochemical testing strips for eco-friendly point of care testing, RSC Advances 10(12) (2020) 7241-7250.

[187] H. Yang, L. Wen, X. Wang, J. Zhao, J. Dong, X. Yin, F. Xu, M. Yang, D. Huo, C. Hou, A test strip electrochemical disposable by 3D MXA/AuNPs DNA-circuit for the detection of miRNAs, Microchimica Acta 189(1) (2022) 1-10.

[188] J. Zhao, C. He, W. Wu, H. Yang, J. Dong, L. Wen, Z. Hu, M. Yang, C. Hou, D. Huo, MXene-MoS2 heterostructure collaborated with catalyzed hairpin assembly for label-free electrochemical detection of microRNA-21, Talanta 237 (2022) 122927.

[189] P. Mahmoodi, M. Rezayi, E. Rasouli, A. Avan, M. Gholami, M. Ghayour Mobarhan, E. Karimi, Y. Alias, Early-stage cervical cancer diagnosis based on an ultra-sensitive electrochemical DNA nanobiosensor for HPV-18 detection in real samples, Journal of nanobiotechnology 18(1) (2020) 1-12.

[190] G. Bolat, Investigation of poly (CTAB-MWCNTs) composite based electrochemical DNA biosensor and interaction study with anticancer drug Irinotecan, Microchemical Journal 159 (2020) 105426.

[191] H.A. Javar, Z. Garkani-Nejad, G. Dehghannoudeh, H. Mahmoudi-Moghaddam, Development of a new electrochemical DNA biosensor based on Eu3+− doped NiO for determination of amsacrine as an anti-cancer drug: Electrochemical, spectroscopic and docking studies, Analytica Chimica Acta 1133 (2020) 48-57.

[192] E.A. Chiticaru, L. Pilan, M. Ioniţă, Electrochemical Detection Platform Based on RGO Functionalized with Diazonium Salt for DNA Hybridization, Biosensors 12(1) (2022) 39.

[193] E. Gasperskaja, V. Kučinskas, The most common technologies and tools for functional genome analysis, Acta Medica Lituanica 24(1) (2017) 1.

[194] Y.-A. Ko, K. Susztak, Epigenomics: the science of no-longer-junk DNA. Why study it in chronic kidney disease?, Seminars in nephrology, Elsevier, 2013, pp. 354-362.

[195] K.-H. Liang, Bioinformatics for biomedical science and clinical applications, Elsevier2013.

[196] M. Tyers, M. Mann, From genomics to proteomics, Nature 422(6928) (2003) 193-197.

[197] W.C. Cho, Proteomics technologies and challenges, Genomics, proteomics & bioinformatics 5(2) (2007) 77-85.

[198] N. Tretyakova, P.W. Villalta, S. Kotapati, Mass spectrometry of structurally modified DNA, Chemical reviews 113(4) (2013) 2395-2436.

[199] J. Guo, R.J. Turesky, Emerging technologies in mass spectrometry-based DNA adductomics, High-throughput 8(2) (2019) 13.

[200] S. Balbo, R.J. Turesky, P.W. Villalta, DNA adductomics, Chemical research in toxicology 27(3) (2014) 356-366.

[201] S. Balbo, S.S. Hecht, P. Upadhyaya, P.W. Villalta, Application of a high-resolution mass-spectrometry-based DNA adductomics approach for identification of DNA adducts in complex mixtures, Analytical chemistry 86(3) (2014) 1744-1752.

[202] M. Sindelar, G.J. Patti, Chemical discovery in the era of metabolomics, Journal of the American Chemical Society 142(20) (2020) 9097-9105.

[203] Q. Wang, J.-Y. Jing, B.-T. Wang, Highly sensitive SPR biosensor based on graphene oxide and staphylococcal protein a co-modified TFBG for human IgG detection, IEEE Transactions on Instrumentation and Measurement 68(9) (2018) 3350-3357.

[204] C. Liu, Q. Cai, B. Xu, W. Zhu, L. Zhang, J. Zhao, X. Chen, Graphene oxide functionalized long period grating for ultrasensitive label-free immunosensing, Biosensors and Bioelectronics 94 (2017) 200-206.

[205] B. Kaur, S. Kumar, B.K. Kaushik, 2D Materials-Based Fiber Optic SPR Biosensor for Cancer Detection at 1550 nm, IEEE Sensors Journal 21(21) (2021) 23957-23964.

[206] M.S. Rahman, M.S. Anower, L.F. Abdulrazak, Modeling of a fiber optic SPR biosensor employing Tin Selenide (SnSe) allotropes, Results in Physics 15 (2019) 102623.

[207] M.S. Rahman, L.F. Abdulrazak, Utilization of a phosphorene-graphene/TMDC heterostructure in a surface plasmon resonance-based fiber optic biosensor, Photonics and Nanostructures-Fundamentals and Applications 35 (2019) 100711.

[208] H.-M. Kim, J.-H. Park, S.-K. Lee, Fiber optic sensor based on ZnO nanowires decorated by Au nanoparticles for improved plasmonic biosensor, Scientific reports 9(1) (2019) 1-9.

[209] Q. Wu, N. Li, Y. Wang, Y. Xu, S. Wei, J. Wu, G. Jia, X. Fang, F. Chen, X. Cui, A 2D transition metal carbide MXene-based SPR biosensor for ultrasensitive carcinoembryonic antigen detection, Biosensors and Bioelectronics 144 (2019) 111697.

[210] B. Meshginqalam, J. Barvestani, Performance enhancement of SPR biosensor based on phosphorene and transition metal dichalcogenides for sensing DNA hybridization, IEEE sensors Journal 18(18) (2018) 7537-7543.

[211] S. Jia, C. Wang, J. Qian, X. Zhang, H. Cui, Q. Zhang, Y. Tian, N. Hao, J. Wei, K. Wang, An upgraded 2D nanosheet-based FRET biosensor: insights into avoiding background and eliminating effects of background fluctuations, Chemical Communications 58(3) (2022) 467-470.

[212] L. Liang, M. Chen, Y. Tong, W. Tan, Z. Chen, Detection of Mycobacterium Tuberculosis IS6110 gene fragment by fluorescent biosensor based on FRET between two-dimensional metal-organic framework and quantum dots-labeled DNA probe, Analytica Chimica Acta 1186 (2021) 339090.

[213] J. Shi, J. Lyu, F. Tian, M. Yang, A fluorescence turn-on biosensor based on graphene quantum dots (GQDs) and molybdenum disulfide (MoS2) nanosheets for epithelial cell adhesion molecule (EpCAM) detection, Biosensors and Bioelectronics 93 (2017) 182-188.

[214] J. Shi, J. Guo, G. Bai, C. Chan, X. Liu, W. Ye, J. Hao, S. Chen, M. Yang, A graphene oxide based fluorescence resonance energy transfer (FRET) biosensor for ultrasensitive detection of botulinum neurotoxin A (BoNT/A) enzymatic activity, Biosensors and Bioelectronics 65 (2015) 238-244.

[215] L. Cao, L. Cheng, Z. Zhang, Y. Wang, X. Zhang, H. Chen, B. Liu, S. Zhang, J. Kong, Visual and high-throughput detection of cancer cells using a graphene oxide-based FRET aptasensing microfluidic chip, Lab on a Chip 12(22) (2012) 4864-4869.

[216] Z. Dehghani, J. Mohammadnejad, M. Hosseini, A.H. Rezayan, Whole cell FRET immunosensor based on graphene oxide and graphene dot for Campylobacter jejuni detection, Food Chemistry 309 (2020) 125690.

[217] E. Song, D. Cheng, Y. Song, M. Jiang, J. Yu, Y. Wang, A graphene oxide-based FRET sensor for rapid and sensitive detection of matrix metalloproteinase 2 in human serum sample, Biosensors and Bioelectronics 47 (2013) 445-450.

[218] H. Zhang, Y. Wang, D. Zhao, D. Zeng, J. Xia, A. Aldalbahi, C. Wang, L. San, C. Fan, X. Zuo, Universal fluorescence biosensor platform based on graphene quantum dots and pyrene-functionalized molecular beacons for detection of microRNAs, ACS applied materials & interfaces 7(30) (2015) 16152-16156.

[219] M. Zhang, B.-C. Yin, X.-F. Wang, B.-C. Ye, Interaction of peptides with graphene oxide and its application for real-time monitoring of protease activity, Chemical Communications 47(8) (2011) 2399-2401.

[220] J.H. Jung, D.S. Cheon, F. Liu, K.B. Lee, T.S. Seo, A graphene oxide based immuno‐biosensor for pathogen detection, Angewandte Chemie 122(33) (2010) 5844-5847.

[221] P. Alonso-Cristobal, P. Vilela, A. El-Sagheer, E. Lopez-Cabarcos, T. Brown, O. Muskens, J. Rubio-Retama, A. Kanaras, Highly sensitive DNA sensor based on upconversion nanoparticles and graphene oxide, ACS applied materials & interfaces 7(23) (2015) 12422-12429.

[222] Z.S. Qian, X. Shan, L. Chai, J. Ma, J. Chen, H. Feng, A universal fluorescence sensing strategy based on biocompatible graphene quantum dots and graphene oxide for the detection of DNA, Nanoscale 6(11) (2014) 5671-5674.

[223] Y. Liao, X. Zhou, D. Xing, Quantum dots and graphene oxide fluorescent switch based multivariate testing strategy for reliable detection of Listeria monocytogenes, ACS applied materials & interfaces 6(13) (2014) 9988-9996.

[224] X.-J. Xing, X.-G. Liu, Y. He, Y. Lin, C.-L. Zhang, H.-W. Tang, D.-W. Pang, Amplified fluorescent sensing of DNA using graphene oxide and a conjugated cationic polymer, Biomacromolecules 14(1) (2013) 117-123.

[225] S. Pang, Y. Gao, Y. Li, S. Liu, X. Su, A novel sensing strategy for the detection of Staphylococcus aureus DNA by using a graphene oxide-based fluorescent probe, Analyst 138(9) (2013) 2749-2754.

[226] Z. Qian, X. Shan, L. Chai, J. Chen, H. Feng, Simultaneous Detection of Multiple DNA Targets by Integrating Dual‐Color Graphene Quantum Dot Nanoprobes and Carbon Nanotubes, Chemistry–A European Journal 20(49) (2014) 16065-16069.

[227] J. Shi, C. Chan, Y. Pang, W. Ye, F. Tian, J. Lyu, Y. Zhang, M. Yang, A fluorescence resonance energy transfer (FRET) biosensor based on graphene quantum dots (GQDs) and gold nanoparticles (AuNPs) for the detection of mecA gene sequence of Staphylococcus aureus, Biosensors and Bioelectronics 67 (2015) 595-600.

[228] Q. Wang, W. Wang, J. Lei, N. Xu, F. Gao, H. Ju, Fluorescence quenching of carbon nitride nanosheet through its interaction with DNA for versatile fluorescence sensing, Analytical chemistry 85(24) (2013) 12182-12188.

[229] K. Hu, T. Zhong, Y. Huang, Z. Chen, S. Zhao, Graphitic carbon nitride nanosheet-based multicolour fluorescent nanoprobe for multiplexed analysis of DNA, Microchimica Acta 182(5) (2015) 949-955.

[230] Q. Xi, D.-M. Zhou, Y.-Y. Kan, J. Ge, Z.-K. Wu, R.-Q. Yu, J.-H. Jiang, Highly sensitive and selective strategy for microRNA detection based on WS2 nanosheet mediated fluorescence quenching and duplex-specific nuclease signal amplification, Analytical chemistry 86(3) (2014) 1361-1365.

[231] D. Bahari, B. Babamiri, A. Salimi, A. Rashidi, Graphdiyne/graphene quantum dots for development of FRET ratiometric fluorescent assay toward sensitive detection of miRNA in human serum and bioimaging of living cancer cells, Journal of Luminescence 239 (2021) 118371.

[232] T.-Y. Chen, P.T.K. Loan, C.-L. Hsu, Y.-H. Lee, J.T.-W. Wang, K.-H. Wei, C.-T. Lin, L.-J. Li, Label-free detection of DNA hybridization using transistors based on CVD grown graphene, Biosensors and Bioelectronics 41 (2013) 103-109.

[233] S. Xu, J. Zhan, B. Man, S. Jiang, W. Yue, S. Gao, C. Guo, H. Liu, Z. Li, J. Wang, Real-time reliable determination of binding kinetics of DNA hybridization using a multi-channel graphene biosensor, Nature communications 8(1) (2017) 1-10.

[234] S. Xu, S. Jiang, C. Zhang, W. Yue, Y. Zou, G. Wang, H. Liu, X. Zhang, M. Li, Z. Zhu, Ultrasensitive label-free detection of DNA hybridization by sapphire-based graphene field-effect transistor biosensor, Applied Surface Science 427 (2018) 1114-1119.

[235] M.T. Hwang, P.B. Landon, J. Lee, D. Choi, A.H. Mo, G. Glinsky, R. Lal, Highly specific SNP detection using 2D graphene electronics and DNA strand displacement, Proceedings of the National Academy of Sciences 113(26) (2016) 7088-7093.

[236] H.-Y. Park, S.R. Dugasani, D.-H. Kang, G. Yoo, J. Kim, B. Gnapareddy, J. Jeon, M. Kim, Y.J. Song, S. Lee, M-DNA/transition metal dichalcogenide hybrid structure-based bio-FET sensor with ultra-high sensitivity, Scientific reports 6(1) (2016) 1-9.

[237] J. Liu, X. Chen, Q. Wang, M. Xiao, D. Zhong, W. Sun, G. Zhang, Z. Zhang, Ultrasensitive monolayer MoS2 field-effect transistor based DNA sensors for screening of down syndrome, Nano letters 19(3) (2019) 1437-1444.

[238] Y. Xia, Y. Sun, H. Li, S. Chen, T. Zhu, G. Wang, B. Man, J. Pan, C. Yang, Plasma treated graphene FET sensor for the DNA hybridization detection, Talanta 223 (2021) 121766.

[239] B. Cai, S. Wang, L. Huang, Y. Ning, Z. Zhang, G.-J. Zhang, Ultrasensitive label-free detection of PNA–DNA hybridization by reduced graphene oxide field-effect transistor biosensor, ACS nano 8(3) (2014) 2632-2638.

[240] J. Lee, P. Dak, Y. Lee, H. Park, W. Choi, M.A. Alam, S. Kim, Two-dimensional layered MoS2 biosensors enable highly sensitive detection of biomolecules, Scientific reports 4(1) (2014) 1-7.

[241] G. Yoo, H. Park, M. Kim, W.G. Song, S. Jeong, M.H. Kim, H. Lee, S.W. Lee, Y.K. Hong, M.G. Lee, Real-time electrical detection of epidermal skin MoS2 biosensor for point-of-care diagnostics, Nano Research 10(3) (2017) 767-775.

[242] H. Park, G. Han, S.W. Lee, H. Lee, S.H. Jeong, M. Naqi, A. AlMutairi, Y.J. Kim, J. Lee, W.-j. Kim, Label-free and recalibrated multilayer MoS2 biosensor for point-of-care diagnostics, ACS applied materials & interfaces 9(50) (2017) 43490-43497.

[243] N. Gao, T. Gao, X. Yang, X. Dai, W. Zhou, A. Zhang, C.M. Lieber, Specific detection of biomolecules in physiological solutions using graphene transistor biosensors, Proceedings of the National Academy of Sciences 113(51) (2016) 14633-14638.
